# Mechanism of CK2 Inhibition by a Ruthenium-Based Polyoxometalate

**DOI:** 10.3389/fmolb.2022.906390

**Published:** 2022-06-02

**Authors:** Simone Fabbian, Gabriele Giachin, Massimo Bellanda, Christian Borgo, Maria Ruzzene, Giacomo Spuri, Ambra Campofelice, Laura Veneziano, Marcella Bonchio, Mauro Carraro, Roberto Battistutta

**Affiliations:** ^1^ Department of Chemical Sciences, University of Padova, Padova, Italy; ^2^ CNR Institute of Biomolecular Chemistry, University of Padova, Padova, Italy; ^3^ Department of Biomedical Sciences, University of Padova, Padova, Italy; ^4^ CNR Institute of Neurosciences, University of Padova, Padova, Italy; ^5^ Institute on Membrane Technology (ITM)-CNR, University of Padova, Padova, Italy

**Keywords:** CK2, inhibition, polyoxometalate, SAXS, substrate competitive

## Abstract

CK2 is a Ser/Thr protein kinase involved in many cellular processes such as gene expression, cell cycle progression, cell growth and differentiation, embryogenesis, and apoptosis. Aberrantly high CK2 activity is widely documented in cancer, but the enzyme is also involved in several other pathologies, such as diabetes, inflammation, neurodegeneration, and viral infections, including COVID-19. Over the last years, a large number of small-molecules able to inhibit the CK2 activity have been reported, mostly acting with an ATP-competitive mechanism. Polyoxometalates (POMs), are metal-oxide polyanionic clusters of various structures and dimensions, with unique chemical and physical properties. POMs were identified as nanomolar CK2 inhibitors, but their mechanism of inhibition and CK2 binding site remained elusive. Here, we present the biochemical and biophysical characterizing of the interaction of CK2α with a ruthenium-based polyoxometalate, [Ru_4_(μ-OH)_2_(μ-O)_4_(H_2_O)_4_ (γ-SiW_10_O_36_)_2_]^10−^ (Ru_4_POM), a potent inhibitor of CK2. Using analytical Size-Exclusion Chromatography (SEC), Isothermal Titration Calorimetry (ITC), and SAXS we were able to unravel the mechanism of inhibition of Ru_4_POM. Ru_4_POM binds to the positively-charged substrate binding region of the enzyme through electrostatic interactions, triggering the dimerization of the enzyme which consequently is inactivated. Ru_4_POM is the first non-peptide molecule showing a substrate-competitive mechanism of inhibition for CK2. On the basis of SAXS data, a structural model of the inactivated (CK2α)_2_(Ru_4_POM)_2_ complex is presented.

## Introduction

Protein kinase CK2 (previously known as casein kinase 2 or CK2) is a eukaryotic Ser/Thr protein kinase, ubiquitous and extremely conserved throughout evolution (Pinna, 2002; Niefind and Battistutta, 2013). Despite the name, it is a “pseudo” casein kinase since casein is not a physiological substrate (Venerando et al., 2014). Indeed, CK2 phosphorylates hundreds of cellular proteins involved in practically all biological processes (St-Denis and Litchfield, 2009), and a dysregulated phosphorylation level of many of its targets has been associated with diverse human diseases (Borgo et al., 2021b). While some pathological conditions seem related to reduced CK2 activity (Dominguez et al., 2021), usually the CK2 overexpression and the resulting abnormally elevated catalytic activity have been associated to diseases. Cancer is the best-known example of this condition, and the role of CK2 in the strengthening of several oncogenic signals has been extensively described (Duncan and Litchfield, 2008; Ruzzene and Pinna, 2010; Trembley et al., 2010; Ortega et al., 2014; Chua et al., 2017). The pharmacological inhibition of CK2 has been largely pursued, not only for cancer but also for the drug resistant phenotype (Borgo and Ruzzene, 2019), and several inhibitors have been discovered and characterized, bearing variable degrees of efficiency and specificity, recently reviewed in (Qiao et al., 2019; Borgo and Ruzzene, 2021).

In CK2, the *α* catalytic subunit (or its isoform *α*’) and the *β* “regulatory” subunit are associated to form the α_2_β_2_ tetrameric holoenzyme, the prevailing form of CK2 in cells (Niefind and Battistutta, 2013). CK2 is considered “intrinsically active” because the α-subunit does not undergo significant conformational changes between active and inactive conformations (which do not exist actually), as typical for other protein kinases, in which phosphorylation events or interactions with regulatory subunits shuffle the enzyme between the two states (Niefind et al., 2001). Instead, in CK2, the α-subunit is structurally locked in the active conformation, mainly because of the unique N-terminal extension and the presence of the DWG (Asp-Trp-Gly) motif, rather than the conventional DFG (Asp-Phe-Gly) one, at the beginning of the activation loop. Together, these two characteristics block the activation segment in the active conformation, hampering the movements seen in other protein kinases, with the formation of inactive structures. As a consequence, the *α* catalytic subunit is catalytically functional either when isolated or part of the tetrameric enzyme. However, mechanisms for the control of CK2 functions in cell should exist, but they have not been firmly established, yet. It was proposed that it is based on a self-inhibitory oligomerization process of the α_2_β_2_ holoenzyme, which is active in the monomeric form, but inactive when it forms trimers and higher ordered assemblies, where substrate binding is hampered (Lolli et al., 2012; Lolli et al., 2014; Lolli and Battistutta, 2015; Lolli et al., 2017). This explanation takes into account the absence of a truly inactive conformation of the catalytic subunit.

Selectivity is one major issue in the development of the most studied class of kinase inhibitors, the ATP-competitive one. This because of the conservation of the structural features of the ATP-binding sites among kinases and other ATP-binding proteins, and the high concentration of ATP inside the cell, ∼1–10 mM. However, despite these difficulties, effective ATP-competitive kinase inhibitors have been successfully developed, and as of 21 January 2022 there are 70 FDA-approved small molecule protein kinase inhibitors for clinical application (Roskoski, 2021) (http://www.brimr.org/PKI/PKIs.htm). Yet, many of these compounds show unfavorable side effects and are convenient only when conventional treatments of cancers have been ineffective (Montazeri and Bellmunt, 2020).

The class of the ATP-competitive inhibitors is the most explored also for CK2. The crystal structure of CK2α in complex with the anthraquinone emodin was the first showing the details of the interaction between the enzyme and an ATP-competitive inhibitor (Battistutta et al., 2000). The main characteristics of the CK2 ATP-binding site were described some time ago (De Moliner et al., 2003; Battistutta et al., 2007; Mazzorana et al., 2008; Battistutta, 2009). As CK2 is constitutively active, only the active conformation (conventionally called “DFG-in”) can be targeted and therefore only the so-called “type I inhibitors” can be developed (Mazzorana et al., 2008; Battistutta, 2009; Sarno et al., 2011; Niefind and Battistutta, 2013; Atkinson et al., 2021). One of the most studied and used type I ATP-competitive inhibitor is CX-4945 (silmitasertib) (Battistutta et al., 2011). This compound is currently in phase II clinical trials for the treatment of various tumors and was established as Orphan Drug for cholangiocarcinoma in the United States. The selectivity issue in targeting the ATP-binding site is underlined by the fact that even CX-4945, despite its potency and efficacy, is able to inhibit also 12 other kinases with nanomolar activity.

**FIGURE 1:**
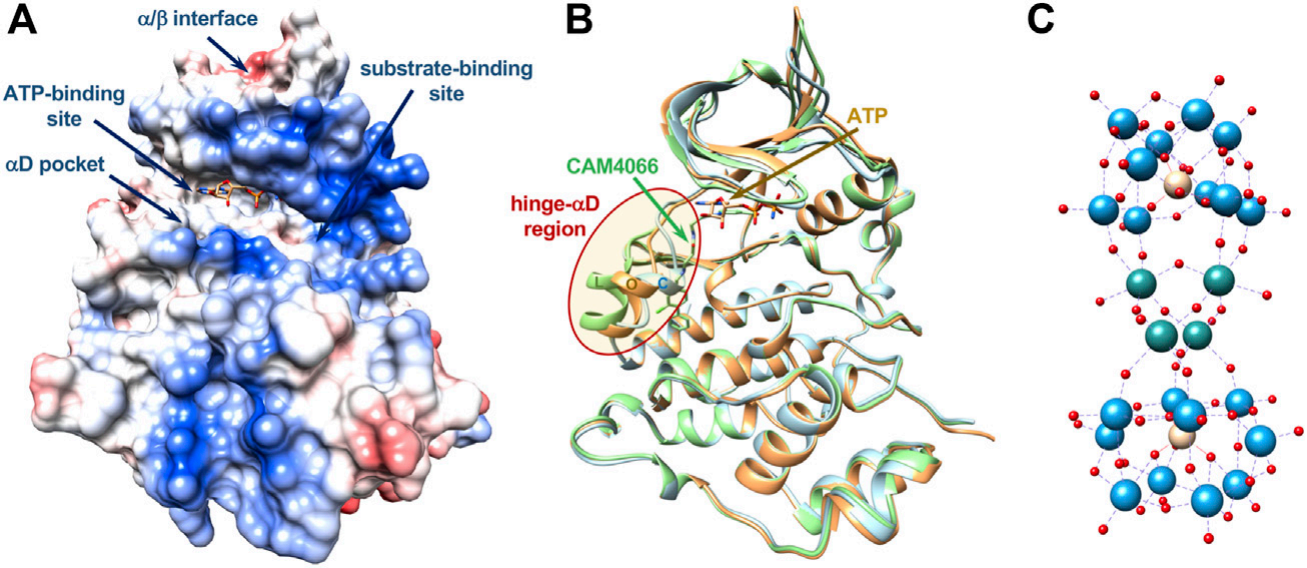
Main interaction sites in catalytic CK2α. **(A)** Surface representation, colored according to electrostatics, of the CK2α-ATP complex (PDB-code 3NSZ); main interactions sites targeted by different classes of inhibitors discussed in the text are shown. Location of allosteric sites are uncertain as well as binding sites for POMs. **(B)** Ribbon representation of three structures of CK2α showing the conformational variability of the very mobile hinge-αD region (red oval), which can adopt a closed (C, in cyan), open (O, in orange) or large (L, in green) conformation (PDB-codes 3Q9W, 3NSZ, and 5CU3, respectively). The αD pocket is created with the “large” conformation, in presence of specific ligands such as CAM4066, shown in green. Bound ATP is shown (carbon atoms in orange). **(C)** Molecular structure of [Ru_4_(H_2_O)_4_(μ-O)_4_(μ-OH)_2_(γ-SiW_10_O_36_)_2_]^10−^ (Ru_4_POM), object of this study: Ru atoms in sea-green, W atoms in light-blue, O atoms in red, Si atoms in yellow. Dimensions of Ru_4_POM are around 10 × 10 × 18 Å (not in scale with CK2).

To cope with the selectivity issue, recently, other inhibition modes were identified, which target the catalytic CK2α subunit in different sites, namely the CK2α/CK2β interaction site, the substrate binding site and some potential allosteric sites outside of the classic “catalytic box” (Figure 1A) (Iegre et al., 2021). Regarding the targeting of the α/β interaction site, although it could suggest high selectivity, one critical point is that the CK2α subunit is constitutively active and CK2β is assumed to modulate, but not to fully suppress or switch on, the catalytic activity of the enzyme. CK2β can also regulate the substrate specificity of CK2 (Pinna, 2002; Borgo et al., 2021a). Furthermore, the binding pocket at the α/β interface, located in the N-terminal domain of the *α* subunit, is shallow and relatively small, and it seems quite difficult to be targeted with efficiency and potency either by peptides or small molecules. Thus, targeting the CK2α/CK2β interaction site does not seem a promising strategy valid for all substrates and for the full inhibition of the catalytic activity of CK2. The development of substrate-competitive molecules (peptides or peptidomimetics) appears to be a promising approach particularly regarding the selectivity issue. In fact, unlike most protein kinases, CK2 exclusively phosphorylates acidic substrates, with a minimum consensus sequence S/T-X-X-E/D/pX, often with acidic residues also in position *n* + 1 and/or *n* + 2 (Pinna, 2002). Polyglutamyl peptides were early identified as CK2 substrate-competitive inhibitors (Meggio et al., 1983). However, the lack of structural data on complexes between CK2 and substrate makes the design of effective peptide inhibitors quite difficult, along with the fact that peptide-based molecules have low pharmacological profiles.

For some compounds targeting CK2 it was proposed an allosteric mechanism of inhibition (Prudent and Cochet, 2009; Iegre et al., 2021), yet the exact mode of action was never clarified and no structural data supporting the allosteric mechanism was produced (Brear et al., 2020).

It was early noted that in the human enzyme the hinge/αD region, located near the active site, has an unusual mobility, unique among protein kinases, with the existence of a closed and an open conformation (Figure 1B) (Battistutta and Lolli, 2011; Papinutto et al., 2012; Niefind and Battistutta, 2013). It was then anticipated that this feature could be exploited for the development of more selective ATP-competitive inhibitors (Battistutta and Lolli, 2011; Papinutto et al., 2012). Indeed, recently, it was found that inhibitor SRPIN803-rev binds to the ATP-binding site of CK2α interacting with the open hinge/αD conformation of CK2α, with a binding mode incompatible with the closed conformation adopted by most protein kinases. This rationalizes the high selectivity of derivatives of SRPIN803-rev when tested on a panel of 320 kinases, despite the not exceptional IC_50_, which ranges from 0.28 to 1.37 μM. Notably, the lead SRPIN803-rev derivative shows an efficacy analogous to that of CX-4945 in cells (Dalle Vedove et al., 2020).

The high mobility of the hinge/αD region, unique to CK2, is at the basis of the very interesting discovery of a new pocket near the ATP-binding site, in the C-terminal domain, the so-called “αD pocket,” corresponding to a “large” conformation of the hinge/αD region (Figure 1B). The αD pocket can be efficiently targeted by a new class of inhibitors, for instance compound CAM4066, which shows an IC_50_ of 0.37 μM, similar to SRPIN803-rev derivatives, and a promising selectivity on a panel of selected 52 kinases (Brear et al., 2016; Iegre et al., 2018).

Polyoxometalates (POMs) are other interesting compounds explored as CK2 inhibitors. POMs are a class of polynuclear oxo-bridged transition metal complexes, which have received extensive attention due to rich topology and tunable chemical/physical properties. In addition to the application in material design (Long et al., 2010) and catalysts (Wang and Yang, 2015), their biological activity has been highlighted (Van Rompuy and Parac-Vogt, 2019). The main advantages of POMs are that their shape, acidity, surface charge distribution and redox potentials can be easily tuned to optimize the interaction with biological macromolecules. POMs were thus proved to cross cell membranes, to display anticancer, antiviral or antibacterial properties. However, their low stability at neutral pH, associated with low selectivity and relevant cytotoxicity has hampered their clinical applications. In order to overcome these drawbacks, POMs can be encapsulated into different delivery systems (Croce et al., 2019). Moreover, their structure can be successfully strengthened by introducing different transition metals (such as Ru, Ti or Co) or by the covalent grafting of organic pendants (Bijelic et al., 2019). In this way, biomedical studies could be performed on POMs stable in solution, employing physiological conditions and concentrations (Čolović et al., 2020). The derivatization of POMs may increase both stability and bioavailability, while providing interesting opportunities for tracking and targeting (Zamolo et al., 2018; Ramezani-Aliakbari et al., 2021; Tagliavini et al., 2021).

Owing to their nano-size, POMs are able to establish various types of interactions with peptides and proteins, ranging from electrostatic interaction and hydrogen bonds, also mediated by water or cations, to other weaker types of interactions (Arefian et al., 2017). These interactions may become more important when organic counterions and appended organic molecules are also present (Zamolo et al., 2018; Tagliavini et al., 2021). POMs are also able to inhibit several enzymes (Zhao et al., 2020). Some POMs were shown to inhibit CK2 in the low nanomolar range (Prudent et al., 2008), with a mechanism of action still unclear, presumably different form that of classical small organic molecules due to their different chemical nature.

Here, we present structural and functional data regarding the CK2 inhibition by a ruthenium-based polyoxometalate, [Ru_4_(H_2_O)_4_(μ-O)_4_(μ-OH)_2_(γ-SiW_10_O_36_)_2_]^10−^ (Ru_4_POM) (Figure 1C), revealing for the first time the mechanism of action of a member of the class of the POMs inhibitors. Ru_4_POM has a significant biomimetic activity, fostered by the ruthenium atoms (Bonchio et al., 2019; Gobbo et al., 2020), and it has been selected for its high stability and solubility in water, even with high ionic strength, associated with a low toxicity. Moreover, its elongated structure results into dimensions (around 10 × 10 × 18 Å) which seem favorable for a better interaction. Indeed, Keggin-type structures, with smaller dimension (10 Å as maximum dimension), show low inhibitory efficiency, unless organic pendants are present on the surface (Prudent et al., 2008).

## Results

### Polyoxometalate Synthesis and Characterization

Na_10_[Ru_4_(H_2_O)_4_(μ-O)_4_(μ-OH)_2_(γ-SiW_10_O_36_)_2_], Ru_4_POM (Supplementary Figure S1), was prepared following a published procedure (Galiano et al., 2021). In order to guarantee its use in all conditions explored, its stability was confirmed by UV-vis, in buffered/saline aqueous environments, up to pH 8.5.

Since the presence of organic ligand as biotin was reported to be potentially useful for CK2 inhibition (Prudent et al., 2008), we also included in the screening some representative hybrid organic-inorganic POMs (Supplementary Figures S1, S2): [γ-SiW_10_O_36_{(C_5_H_7_N_2_OS)(CH_2_)_4_CONH(CH_2_)_3_Si}_2_O]^4−^, Biotin-SiW_10_ (Zamolo et al., 2018) and [γ-SiW_10_O_36_{(C_16_H_9_)SO_2_NH(CH_2_)_3_Si}_2_O]^4−^, Trp-SiW_10_ (Syrgiannis et al., 2019), which were prepared as tetrabutyl ammonium (TBA) salts, following post-functionalization strategies of the Keggin POM K_8_[γ-SiW_10_O_36_]. The corresponding sodium salts were prepared by counterion exchange as previously described (Zamolo et al., 2018). In case of Trp-SiW_10_, the two enantiomeric forms of tryptophan were investigated. The giant-wheel-shaped (NH_4_)_12_[Mo_36_(NO)_4_O_108_(H_2_O)_16_], Mo_36_POM, was also prepared (Amini et al., 2015) to evaluate the impact of the dimensions and shape.

### *In Vitro* CK2 Inhibition by Ru_4_POM

First, we analyzed the efficacy of different POMs in decreasing the catalytic activity of recombinant CK2 (Table 1). We found that all polyoxotungstates inhibited CK2α, with similar IC_50_ in the low nM range, however, the most powerful compound was Ru_4_POM (IC_50_ 3.63 nM). Concerning the hybrid polyoxotungstates, we observed a moderately higher activity for the lipophilic TBA salts. In addition, both the nature and the configuration of the organic ligands had an impact on the inhibitory effect. The larger polyoxomolybdate showed very weak activity on CK2. Owing to these results, we focused the rest of our study on Ru_4_POM.

**TABLE 1:** Inhibitory activity of different POMs.

POM compound	IC_50_ (nM)		
	FL-CK2α	CK2α	α2β2
Mo_36_POM	>1,000	—	—
Na (L)Trp-SiW_10_	18.2 ± 1.9	—	—
Na (D)Trp-SiW_10_	12.8 ± 1.7	—	—
Na Biotin-SiW_10_	16.4 ± 1.3	—	—
TBA (L)Trp-SiW_10_	8.5 ± 0.4	—	—
TBA (D)Trp-SiW_10_	11.0 ± 0.6	—	—
TBA Biotin-SiW_10_	5.3 ± 0.6	—	—
Ru_4_POM	3.63 ± 0.17	3.63 ± 0.09	5.57 ± 0.68

IC_50_ values were determined for CK2 inhibition by means of radioactive kinase assays using the synthetic peptide CK2-tide as substrate, in the presence of increasing concentrations of the indicated POM compound. For Ru_4_POM, besides full-length CK2α (FL-CK2α), a C-terminal truncated form of CK2α (CK2α) and the tetrameric holoenzyme (α_2_β_2_) were tested. The other POMs were tested only on full-length CK2α. At least two independent experiments were performed. Mean values ± SEM are reported.

Most inhibition assays and all the structural studies were performed with CK2α deleted of the last 55 C-terminal residues since this deletion confers higher stability to the protein without affecting the catalytic activity (Niefind et al., 2000). Indeed, we found that the full-length monomeric enzyme (FL-CK2α) displayed identical sensitivity to Ru_4_POM (Table 1). Moreover, the presence of the *β* subunit only marginally reduced the efficacy of Ru_4_POM, the tetrameric enzyme displaying an IC_50_ of 5.57 nM (Table 1).

**FIGURE 2:**
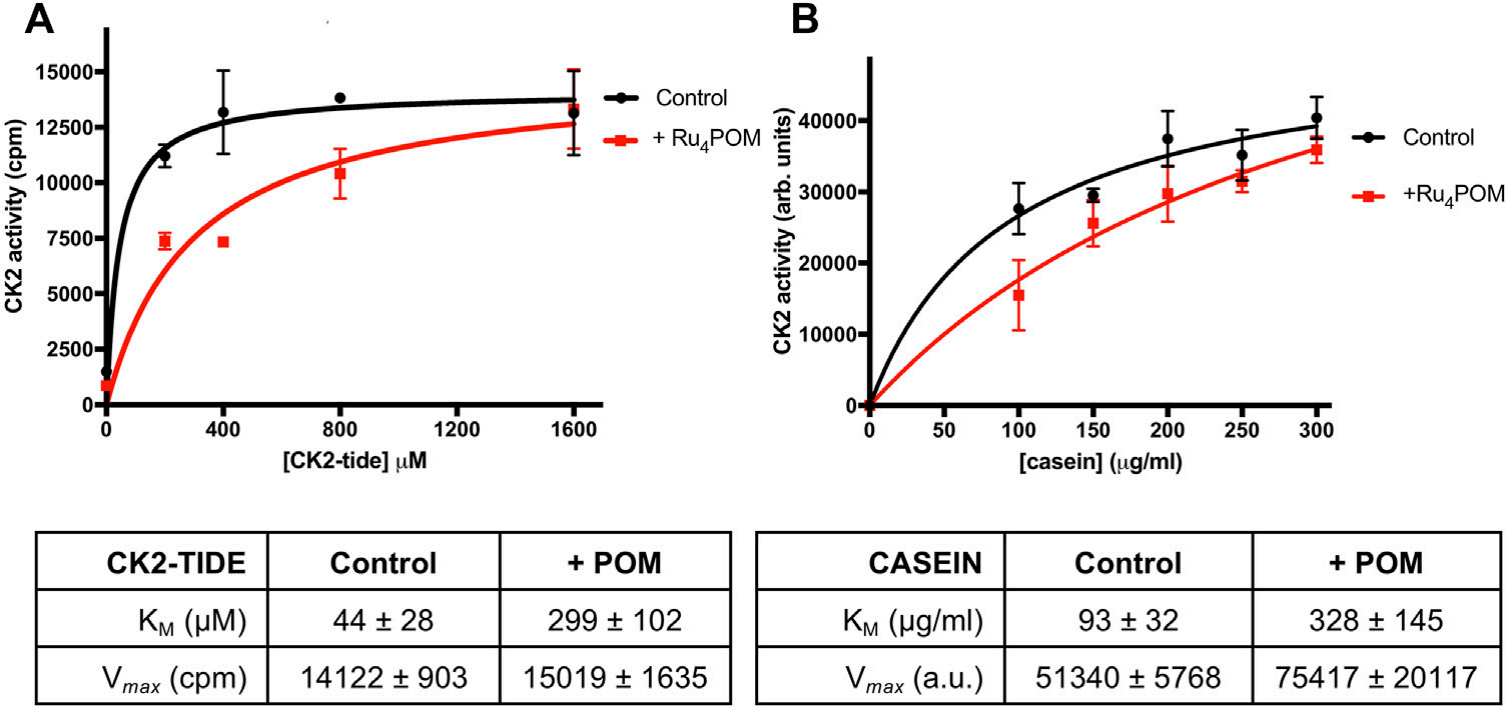
Substrate-competitive inhibition. **(A)** CK2 activity was measured by radioactive phosphorylation assays, with increasing concentrations of CK2-tide peptide, in the absence or presence of 5 nM Ru_4_POM. Radioactivity was detected by scintillation counting (cpm). **(B)** CK2 activity was measured by radioactive phosphorylation assays, with increasing concentrations of casein, in the absence or presence of 18 nM Ru_4_POM. Radioactivity was detected by digital autoradiography (arbitrary units). At least two independent experiments were performed. Mean values ± SEM are reported in the graphs; the tables on the bottom report the kinetics parameters ± SEM calculated by Michaelis-Menten analysis.

We then evaluated if the Ru_4_POM inhibition efficacy depends on the kind of substrate. We observed a slightly lower inhibition when casein replaced the CK2-tide peptide as substrate; the IC_50_, however, was still in the low nM range (18.50 ± 1.04 nm). We then checked whether Ru_4_POM competes with the substrates for the CK2 binding-site. As shown by the kinetics analysis reported in Figure 2, we found that Ru_4_POM exerts a competitive inhibition towards both model substrates, since it reduced the affinity (increased K_M_ values for both CK2-tide peptide and casein), without decreasing the V_max_ values.

**FIGURE 3:**
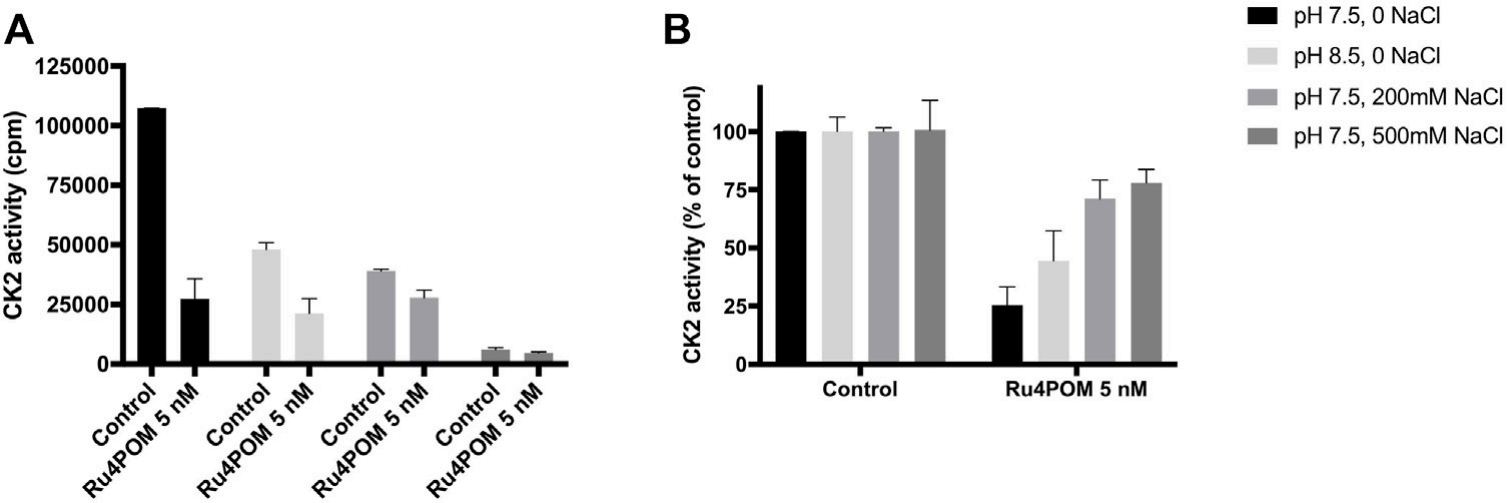
Effect of pH and ionic strength. CK2 activity was measured by radioactive phosphorylation assays towards CK2-tide peptide, in the absence or presence of 5 nM Ru_4_POM, at different conditions of pH and NaCl concentration, as indicated. Radioactivity was detected by scintillation counting (cpm) **(A)**. In **(B)**, 100% activity was assigned to each control, measured in the absence of Ru_4_POM. Mean values ± SEM of at least two independent experiments are reported.

In the experiments described so far, the activity of recombinant CK2α was evaluated at the optimal *in vitro* conditions, namely at pH 7.5 and in the absence of NaCl. Given the high negative charge of Ru_4_POM, we evaluated if the inhibitory efficacy was affected by changes in pH and ionic strength. Deviation from the optimal conditions reduced the activity of the controls (see samples without Ru_4_POM in Figure 3A). Nonetheless, the residual activity was sufficient to perform the experiments, which show that the percent of inhibition induced by Ru_4_POM was reduced by the pH change: at 5 nM Ru_4_POM, the inhibition dropped from 75% at pH 7.5% to 66% at pH 8.5 (Figure 3B). A higher variation in the inhibition values was seen varying the ionic strength, from 75% inhibition observed without NaCl to 29% and 22% in presence of NaCl at 0.2 M or 0.5 M, respectively.

### Ru_4_POM Inhibits Endo-Cellular CK2

**FIGURE 4:**
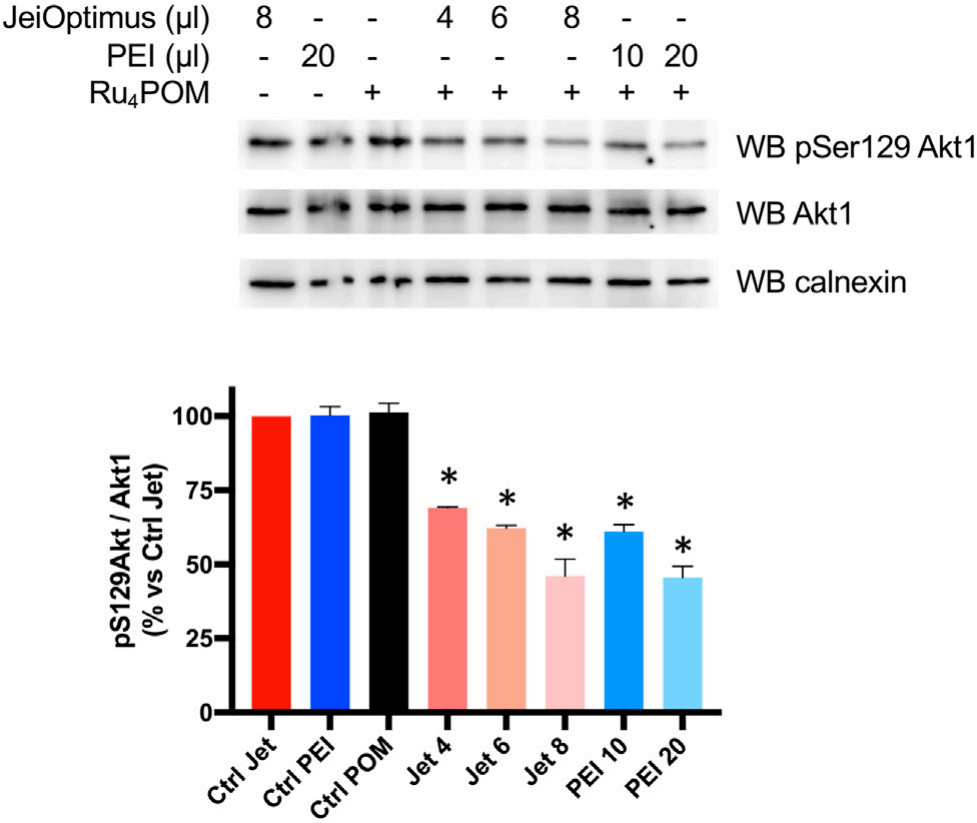
Cellular activity of Ru_4_POM. HEK-293T cells were treated for 24 h as indicated. Ru_4_POM was used at 10 μM. 20 μg protein from total cell lysates were analyzed by SDS-PAGE and Western blot (WB) with the indicated antibodies. Calnexin was used as loading control. Quantitation of the signal is shown on the bottom panel (mean values ± SEM of two independent experiments). *p < 0.05.

Next, we wanted to assess whether Ru_4_POM was able to inhibit CK2 inside intact cells. Preliminary attempts to detect inhibition of CK2 activity in Ru_4_POM-treated cells failed, indicating a too small cell permeability of the compound (not shown). Therefore, we tried to treat cells with Ru_4_POM in combination with increasing amounts of two different cationic transfection reagents, the stable cationic polymer polyethylenimine (PEI) and the cationic liposome JetOptimus (Figure 4). Endogenous CK2 activity was monitored by means of the phospho-antibody towards Akt1 phospho-Ser129, a well-known and specific target of CK2 (Ruzzene et al., 2010). We observed that cell treatment with Ru_4_POM combined with one of the cationic compounds reduced the phosphorylation of this site (while the total amount of Akt1 protein was unchanged) and the effect was potentiated by increasing the amount of the transfecting reagents, likely due to higher amounts of Ru_4_POM delivered into the cells. These results indicate that Ru_4_POM is suitable for CK2 inhibition in cell, as it can be transferred inside intact cells, and that it is able to target endogenous CK2.

### Interaction Between CK2α and Ru_4_POM by Analytical Size-Exclusion Chromatography

Analytical Size Exclusion Chromatography was then used to investigate the direct interaction between Ru_4_POM and CK2α *in vitro*. SEC elution profiles (Supplementary Figure S3) were monitored at 280 nm, where both protein and Ru_4_POM have an absorption contribution (blue and black curve, respectively), but also at 500 nm, where only the signal of Ru_4_POM is present (red curve). The lack of absorption of CK2α at 500 nm (green curve) allows to unequivocally recognize the elution of Ru_4_POM. In 25 mM Tris, 500 mM NaCl, 1 mM DTT, pH = 8.5, free CK2α elutes at 12.6 ml retention volume, in the monomeric form (Supplementary Figure S3, blue curve), in accordance with the well-known notion that CK2α is monomeric at high salt, typically above 0.4 M NaCl, and tends to aggregate at lower ionic strengths (Niefind et al., 1998; Ermakova et al., 2003; Seetoh et al., 2016), as also confirmed by our SEC-SAXS experiments (see below). In the same running conditions, Ru_4_POM elutes at higher elution volumes, at 16.0 ml (Supplementary Figure S3, black curve at 280 and red curve at 500 nm), corresponding to lower hydrodynamic volumes, in accordance with the smaller dimensions (MW 5 kDa).

**FIGURE 5:**
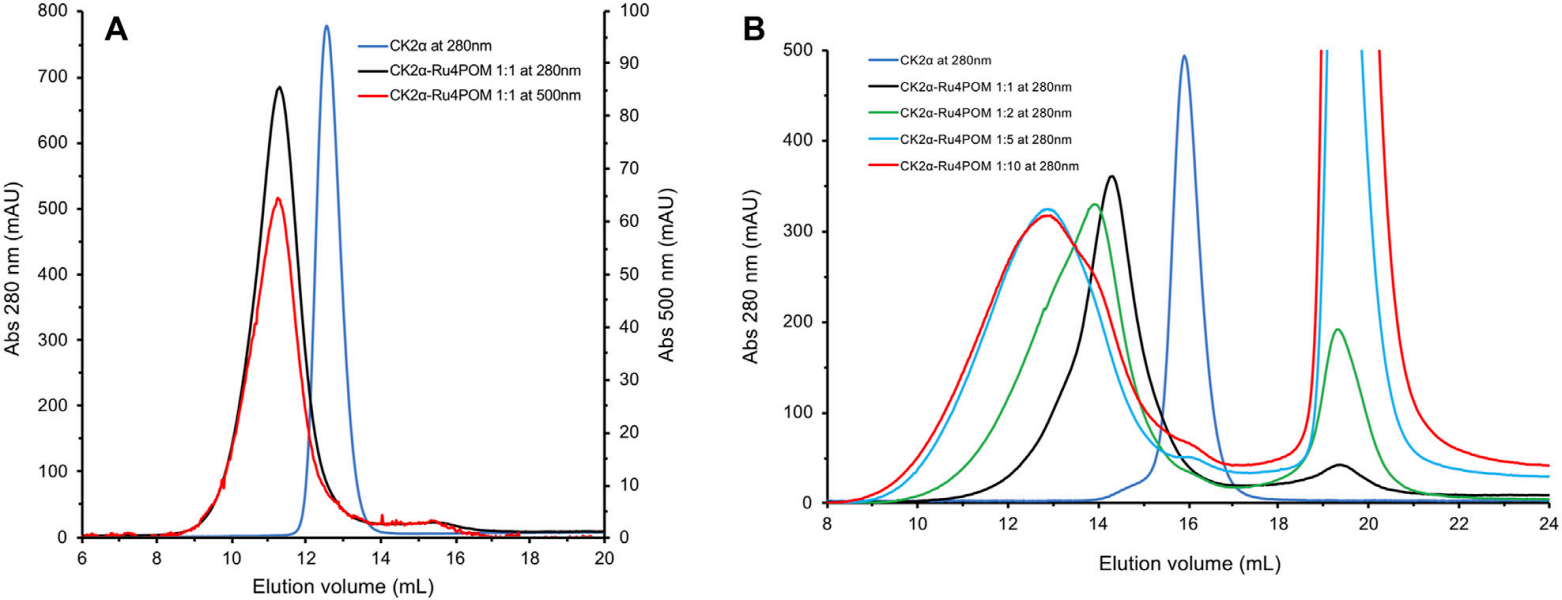
SEC elution profiles of Ru_4_POM/CK2α mixtures at different molar rations. **(A)** In black, the elution profile recorded at 280 nm of a 1:1 mixture of Ru_4_POM:CK2α, in 25 mM Tris, 500 mM NaCl, 1 mM DTT, pH = 8.5, on a 10/30 Superdex 75 GL column. On the basis of the calibration curve of the column and on the comparison with monomeric CK2α (blue curve) the black curve corresponds to the dimeric form of the enzyme. The perfect superposition of the peaks recorded at 280 and 500 nm (red curve) indicates the co-elution of CK2α and Ru_4_POM and hence the formation of a stable complex. The absence of peaks corresponding to the single species suggests that the complex eluting at 11.3 ml has a stoichiometry (CK2α)_2_(Ru_4_POM)_2_. **(B)** Experiments were performed on a 10/30 Superdex 200 GL column, in 25 mM Tris, 500 mM NaCl, 1 mM DTT, pH = 8.5, followed at 280 nm. In blue and black, elution profiles of free monomeric CK2α and Ru_4_POM:CK2α in 1:1 M ratio, respectively. Increasing amounts of Ru_4_POM induce the formation of larger oligomers, as evident by the lower elution volume of the peaks corresponding to molar rations 2:1 (shown in green) and 5:1 (shown in cyan). The widening of the peaks indicates the co-existence of different oligomeric species. The further increase to 10:1 (in red) does not vary the nature of the oligomeric forms, indicating that the saturation of the oligomerization process is reached at around 5:1 M ratio.

The elution profile of samples with Ru_4_POM:CK2α in a 1:1 M ratio on a Superdex 75 column shows a unique peak at about 11.4 ml elution volume (Figure 5A, black curve), with the disappearance of the peaks corresponding to the isolated species (Figure 5A, blue curve for monomeric CK2α). The presence of the absorption at 500 nm for the peak at 11.4 ml (red curve) confirms that all Ru_4_POM are tightly bound to CK2α, with the formation of a stable complex. From the elution volume it can be estimated that the complex is formed by two molecules of CK2α. This, together with the 1:1 M ratio of Ru_4_POM:CK2α, indicates that a complex of stoichiometry (CK2α)_2_(Ru_4_POM)_2_ is formed.

To further investigate the interaction between CK2α and Ru_4_POM, we analysed samples with increasing molar ratios in a Superdex 200 column, which has a higher MWs resolution range. The elution profiles with the unchanged buffer system, 25 mM Tris, 500 mM NaCl, 1 mM DTT, pH = 8.5, of samples with various Ru_4_POM/CK2α ratios, namely 1:1, 2:1, 5:1, and 10:1, are shown in Figure 5B. It is evident that increasing amounts of Ru_4_POM induce the formation of species with larger hydrodynamic dimensions, that is, high-order oligomeric forms of CK2α/Ru_4_POM complexes. Very similar elution profiles for ratios 5:1 and 10:1 indicate that the saturation is reached for such values. The large variations in the elution volumes are explained by the formation of oligomeric forms of CK2α/Ru_4_POM complexes rather than by the formation of species where monomeric CK2α (MW 40 kDa) is interacting with multiple POMs units (MW 5 kDa). Overall, the SEC experiments indicate that Ru_4_POM induces the oligomerization of CK2α, with the formation of dimers when the ratio is 1:1 and of larger assemblies when the ratio is higher, with the saturation reached around 5:1 ratio.

### Interaction Between CK2α and Ru_4_POM by Isothermal Titration Calorimetry

**FIGURE 6:**
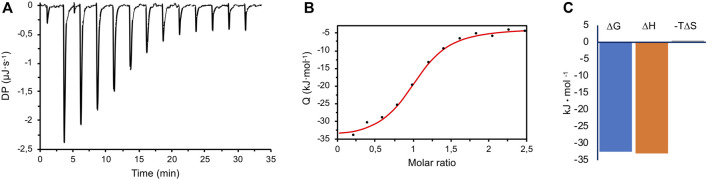
ITC measurements. **(A)** A representative raw titration data (black thermogram) obtained from the titration of 30 μM CK2α with 400 μM Ru_4_POM at 25.3°C, in 25 mM Tris, 500 mM NaCl, pH 8.5. **(B)** Wiseman plot of integrated data (black circles) and fitted isothermal binding curve (in red). From two independent experiments, an apparent mean K_D_ of 2.09 ± 0.36 μM for the single macroscopic dissociation constant was obtained, with a 1:1 stoichiometry. **(C)** The mean ΔG of the overall binding is dominated by the enthalpic term (ΔH), with a negligible entropic contribution (‒TΔS).

To better understand the interaction between CK2α and Ru_4_POM, the thermodynamic parameters of the binding were determined by isothermal titration calorimetry (ITC). In the ITC measurements, CK2α was titrated with Ru_4_POM; both species were in 25 mM Tris, 500 mM NaCl, pH = 8.5. A representative experiment is shown in Figure 6, where the exothermic nature of the interaction is evident. Here, the CK2α/Ru_4_POM titration produces a typical single transition, saturation-shaped thermogram, which can be fitted by the sigmoidal binding isotherm of the one binding-site model. From two independent experiments, we derived an apparent mean K_D_ of 2.09 ± 0.36 μM for the single macroscopic dissociation constant. The derived stoichiometry for the complex is 1:1 (1.04 ± 0.02), in accordance with the chromatographic data, thus supporting the formation of a stable (CK2α)_2_(Ru_4_POM)_2_ complex. The analysis of the thermodynamic signature for the binding of Ru_4_POM to CK2α reveals the dominance of the enthalpic contribution (ΔH = −32.99 ± 1.42 kJ/mol) over a negligible entropic term (−TΔS = 0.50 ± 2.43 kJ/mol), and a final ΔG = −32.48 ± 0.46 kJ/mol (Figure 6C), evidencing the importance of electrostatics and hydrogen-bonds (which can be considered a form of electrostatic interaction) for the binding. For the sake of comparison, Keggin-type POMs as [H_2_W_12_O_40_]^6−^ (H_2_W_12_) interacting with human serum albumin (HSA) were also characterized by enthalpically driven exothermic process, while bigger W-based POMs as [NaP_5_W_30_O_110_]^14−^ (P_5_W_30_), showed an important endothermic component, mainly attributed to the unfolding of the protein (Zhang et al., 2008).

### Interaction Between CK2α and Ru_4_POM by SAXS

**FIGURE 7:**
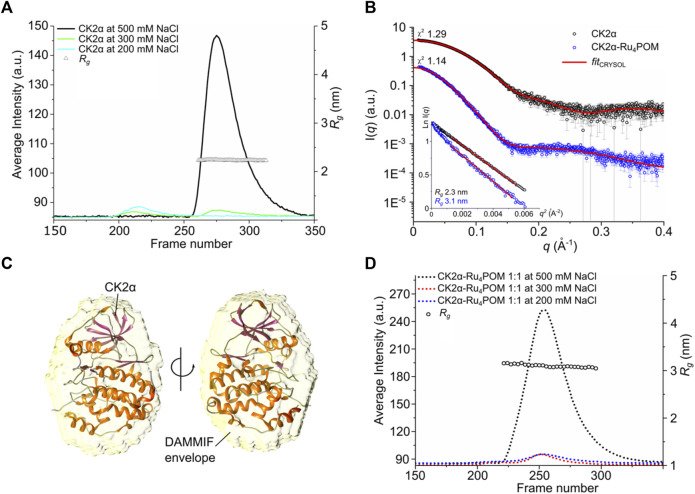
SAXS studies on CK2α-Ru_4_POM complex. **(A)** SEC-SAXS chromatograms of *R*_*g*_ and I(0) traces as functions of frames of separated CK2α, in buffer with 0.5, 0.3, and 0.2 M NaCl (black, green, and cyan lines, respectively). Triangles represent the calculated radius of gyration, *R*_*g*_ in nm, in the selected frames collected in buffer containing 0.5 M NaCl. **(B)** *I*(*q*) versus *q* experimental SAXS profiles for CK2α and CK2α-Ru_4_POM (black and blue circle, respectively) with CRYSOL fit (red lines). χ^2^ values for the CRYSOL fitting are indicated. The curves are shifted by an arbitrary offset for better comparison. In the *inset*, the Guinier fits for the two samples and the calculated radius of gyration are shown. **(C)** CK2α DAMMIF model (yellow envelope shown in transparency) overlaid with the CK2α crystal structure (PDB entry 3q04). **(D)** SEC-SAXS chromatograms of *R*_*g*_ and I(0) traces as functions of frames of separated CK2α-Ru_4_POM complex at 1:1 M ratio and increasing NaCl concentration.

Initially, we tested the protein stability at three different ionic strengths (namely 0.5, 0.3, and 0.2 M NaCl), on a SEC GE Superdex Increase 200 (3.2/300) column, equilibrated with buffer 25 mM Tris pH 8.5. The *R*_*g*_ and I(0) traces as functions of the SEC elution profile frames showed that the protein was monodispersed in presence of 0.5 M NaCl (Figure 7A, black curve). However, decreasing the ionic strength to 0.3 and 0.2 mM NaCl resulted in protein precipitation prior SAXS experiments, as indicated by the very low signals (green and cyan curves), in line with the known behavior of CK2α at low salt concentration.

Primary analysis from selected scattering curves of CK2α at 0.5 M NaCl indicated that the protein is globular, with *R*_*g*_ value of 2.3 nm, corresponding to an estimated molecular weight of about 39 kDa, as expected from monomeric CK2α, in line with the MW estimation from the previous SEC experiments. The comparison between the CK2α crystal structure and the SAXS data showed good fitting value (χ^2^ 1.29) (Figure 7B) and the 3D bead model of the DAMMIF envelope overlaid well the crystal structure of CK2α (Figure 7C), further supporting the presence of the monomeric state at 0.5 M NaCl.

We then analyzed a mixture of CK2α:Ru_4_POM at 1:1 M ratio in presence of 0.5 M NaCl, and the scattering data on the single monodisperse species displayed significant structural differences compared to the free form of CK2α (Figure 7B). Notably, at this concentration Ru_4_POM does not significantly contribute to the scattering profiles (Supplementary Figure S4A). The *R*_*g*_ of the complex determined by Guinier analysis showed a value of 3.1 nm (Figure 7B) and a molecular weight of about 79.7 kDa, a mass that corresponds to the formation of a stable CK2α dimer, in accordance with the previous SEC estimations. This supports our hypothesis, based on previous SEC and ITC data, that CK2α undergoes a Ru_4_POM-mediated dimerization, with the formation of a stable (CK2α)_2_(Ru_4_POM)_2_ complex. Importantly, in accordance with the lack of a significative entropic contribution for the CK2α/Ru_4_POM interaction seen with ITC, SAXS data do not show any evidence of protein denaturation.

Experiments on 1:1 mixtures of CK2α:Ru_4_POM at lower ionic strengths, i.e., 0.3 and 0.2 M NaCl, caused protein precipitation of the sample, as in the case of free CK2α, hampering the possibility of reliable SAXS analyses (Figure 7D).

Next, we monitored the effect of 2 and 10 M equivalent of Ru_4_POM at 0.5 M NaCl, and the *R*_*g*_ and I(0) traces showed the formation of soluble complexes but in a multi-component system, with different size and molecular weight species co-existing in solution (Supplementary Figure S4B). We then tried to get more detailed structural information on the nature of the (CK2α)_2_(Ru_4_POM)_2_ complex from the available SAXS data. Starting from the evidence that Ru_4_POM actively induces the dimerization of CK2α, the most reasonable assumption is that Ru_4_POM structurally mediate the interaction between two molecules of the enzyme. This is the simpler and more plausible explanation that globally takes into account our SEC, ITC and SAXS data.

**FIGURE 8:**
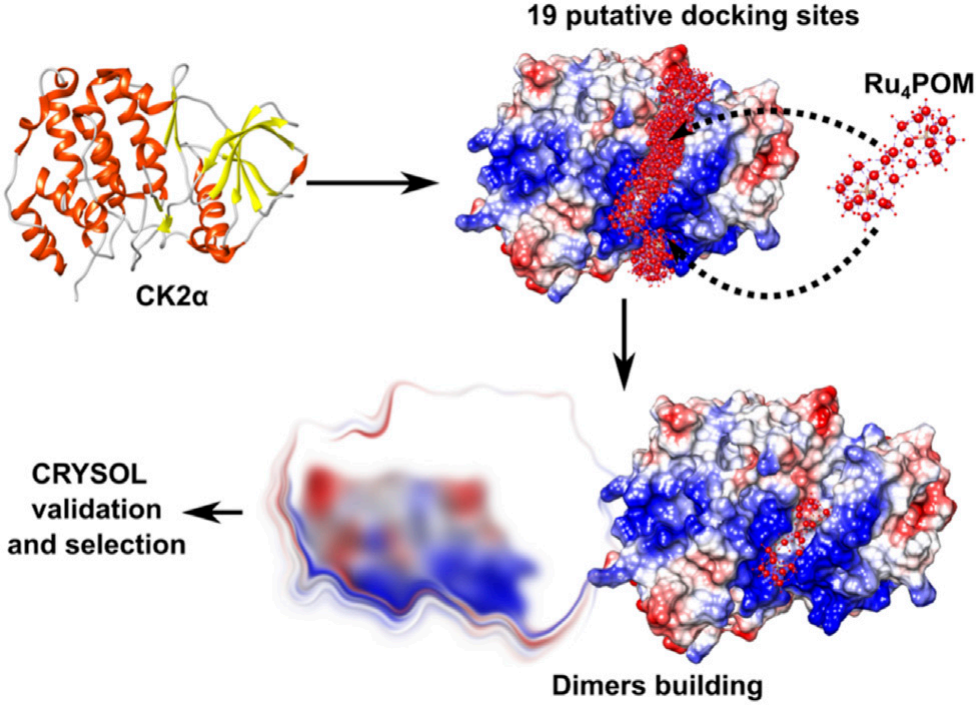
Strategy for modelling the CK2α-Ru_4_POM dimer. Docking of Ru_4_POM to CK2α generated 19 putative CK2α-Ru_4_POM docking models. Each model was processed with Symmdock to obtain 100 possible dimer configurations. Approximately 1,900 dimeric models were validated using CRYSOL. Finally, only dimers stabilized by inter-molecular Ru_4_POM-mediated contacts were selected (“selection”).

Then, we first built possible structural models of the interaction between Ru_4_POM and CK2α, trying to identify the binding site (Figure 8). The docking of Ru_4_POM on monomeric CK2α was obtained using geometry-based molecular docking algorithms (Schneidman-Duhovny et al., 2005) and yielded several models with good molecular shape complementarity, where negatively charged Ru_4_POM invariably interacts with CK2α along the large positively charged area present on the protein surface between the ATP binding pocket and the substrate binding site (Figure 1A). This result is in accordance with the biochemical data that indicates a substrate-competitive mechanism of inhibition, and with the thermodynamic signature of the interaction, largely enthalpic as deduced by ITC measurements. Since the precise location of Ru_4_POM in the substrate-binding site of CK2α is not known, we generated possible dimers starting from each of the 19 plausible CK2α-Ru_4_POM docking models (Figure 8).

**FIGURE 9:**
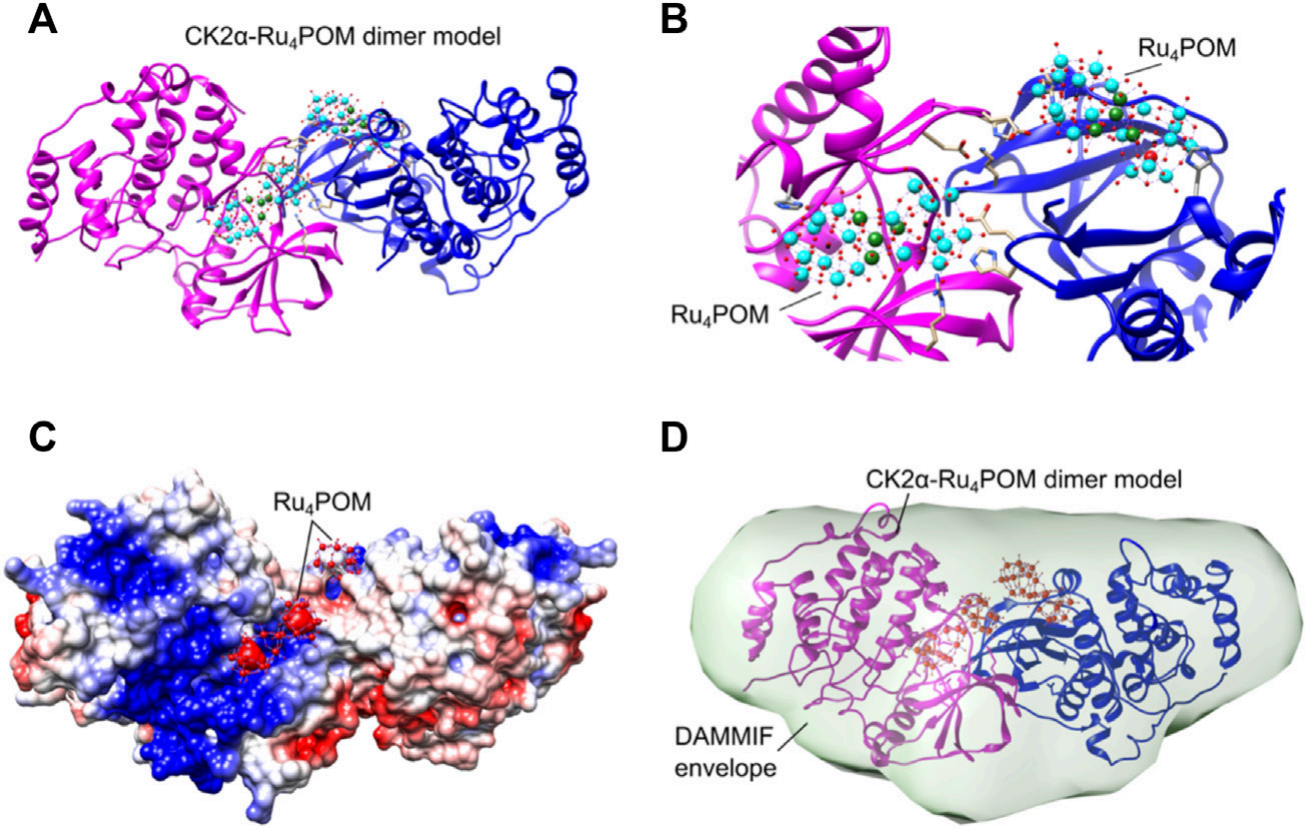
Structural organization of the (CK2α)_2_(Ru_4_POM)_2_ complex. **(A)** Proposed CK2α-Ru_4_POM dimer model showing two interacting CK2α (colored in magenta and blue) and two Ru_4_POM further connecting the two subunits. **(B)** Closer view on the CK2α-Ru_4_POM interface. Spheres colored in cyan represent 10 W atoms, in green 4 Ru atoms, in red O atoms and in black 2 Si atoms. **(C)** Electrostatic surface of the CK2α-Ru_4_POM dimer model. Positive and negative charges are depicted in blue and red, respectively. The potentials were calculated using programs PDB2PQR and APBS (Jurrus et al., 2018). **(D)** CK2α-Ru_4_POM DAMMIF model (light green envelope shown in transparency) overlaid with the proposed CK2α-Ru_4_POM dimer model.

First, the predicted dimers were validated based on the best χ^2^ value obtained fitting the structures with the experimental SAXS data; then, only dimers stabilized by inter-molecular Ru_4_POM-mediated contacts were selected (Figure 8; Supplementary Figure S5). This strategy allowed to identify a CK2α-Ru_4_POM dimeric configuration displaying optimal fitting to SAXS data, i.e., χ^2^ value 1.14 (Figure 9). In this model, each of the two Ru_4_POM molecules bind to each of the two CK2α subunits, in a region adjacent to the ATP binding pocket, between the hinge-αD region and the Gly-rich loop, overlapping part of the substrate-binding site. Ru_4_POM engages both intermolecular electrostatic interactions and H-bonds with polar and positively charged residues of the enzyme. In this (CK2α)_2_(Ru_4_POM)_2_ model direct contacts between the two molecules of CK2α are established. The predicted structural model overlays well with the DAMMIF elongated envelope obtained from CK2α-Ru_4_POM SAXS data analysis (Figure 9D).

## Discussion

It was reported that members of the family of the polyoxometalates are potent inhibitors of the CK2 activity *in vitro*. However, their mechanism of inhibition and CK2 binding site are not firmly established yet. For one of the best POM inhibitors, [P_2_Mo_18_O_62_]^6−^, steady-state kinetic analysis showed that it did not target CK2α either at the ATP-binding site or at the protein substrate binding sites. Site-directed mutagenesis and proteolytic degradation of the CK2α-POM complex suggested that this POM binds to domains containing key structural elements such as the Gly-rich loop, the helix αC and the activation segment. Nevertheless, the low chemical stability in an aqueous environment of this POM hampered to establish a clearly defined binding site and inhibition mechanism (Prudent et al., 2008).

Here, we confirm that different POMs are potent inhibitor of CK2α, with IC_50_ in the low nanomolar range, the best one being a ruthenium-based polyoxometalate, [Ru_4_(μ-OH)_2_(μ-O)_4_(H_2_O)_4_ (γ-SiW_10_O_36_)_2_]^10−^. Some hybrid organic-inorganic POMs belonging to the Keggin decatungstosilicate family, which share the same SiW_10_O_36_ unit of Ru_4_POM, were also shown to be promising. The variation of the inhibitory activity with the organic domains will thus deserve attention, for the possibility to anchor suitable recognition motifs and develop, for example, bi-specific inhibitors (Iegre et al., 2021). Interestingly, cell penetration of these derivative has been previously demonstrated (Zamolo et al., 2018). For now, we have focused our attention on the structurally rigid Ru_4_POM.

Ru_4_POM has an IC_50_ of 3.63 nM on the catalytic subunit, either full-length or deleted of the last 55 C-terminal residues, which are flexible in solution. A very similar IC_50_, 5.57 nM, was found on the tetrameric enzyme. This similarity in the IC_50_ values exclude that the Ru_4_POM binding region is located either at the C-terminus of CK2α or at the α/β interface**.** Importantly, we showed that this compound is stable in the aqueous condition of the biochemical assays (up to pH 8.5), indicating that the full molecule and not some decomposition fragments are active on the enzyme, as instead reported for other POMs (Prudent et al., 2008).

Ru_4_POM has a net negative charge, like the CK2 substrates, therefore we tested whether it works with a substrate-competitive mechanism. Indeed, we show that Ru_4_POM competes with the substrates, either small peptides or casein, indicating that it interacts with CK2 at the level of the substrate-binding site. This interaction is modulated by the pH and the ionic strength, in accordance with a substrate-competitive mechanism of inhibition where the electrostatic interactions play an important role in the binding of both negative substrates and Ru_4_POM to the positively charged CK2 substrate-binding site.

The physical-chemical properties of Ru_4_POM seem to penalize its transport across the cellular membrane of HEK-293T cells, and, in fact, we do not detect any CK2 inhibition on intact cells treated with the free compound, in line with previously reported observations (Prudent et al., 2008). However, when applied in combination with cationic transfection reagents, we do observe inhibition of endo-cellular CK2, meaning that, with the appropriate delivery system, it can be transported inside the cells despite its dimensions and its highly negative charge. It is worth mentioning that CK2 is also present on the outer cell surface [CK2 ecto-kinase (Rodríguez et al., 2005)], with functions that are largely unknown; in this view, Ru_4_POM could represent a valuable tool to discriminate between ecto- and endo-cellular CK2, according to the administration conditions.

To possibly decipher the mechanism of inhibition of Ru_4_POM on CK2 we performed different biophysical analyses. Size-exclusion chromatography experiments at 0.5 M NaCl show that Ru_4_POM induces the dimerization of CK2α, with the formation of a (CK2α)_2_(Ru_4_POM)_2_ complex, with no residual presence of the single components, indicating the formation of a strong and stable complex. SAXS experiments confirm the presence of such a complex, with dimeric CK2α when Ru_4_POM is present in equimolar amount. According to SEC data, larger assemblies are formed at higher molar ratios, with the saturation reached at around 5:1 for Ru_4_POM:CK2α.

The specific interaction between Ru_4_POM and CK2α with the formation of a stable complex is confirmed by ITC measurements, which indicates a macroscopic apparent mean K_D_ of 2.09 ± 0.36 μM and a 1:1 stoichiometry, in accordance with SEC and SAXS data. The observed thermodynamic parameters agree with literature data on the interaction between inorganic POMs and proteins, which are characterized by K_D_ in the μM range (Zhang et al., 2007; Zamolo et al., 2018; Vandebroek et al., 2021) and by a dominant enthalpic contribution. The exothermal process, in particular, entails the key role of the electrostatic interactions and hydrogen bonds, in line with the polar nature of the interaction between Ru_4_POM and the substrate-binding site of CK2. The negligible entropic term, instead, shows that the possible stiffening of the protein structure on one hand, and the dehydration of this highly hydrophilic POM, on the other hand, are not relevant. Noteworthy, despite larger POMs may display higher free energy of binding (Zhang et al., 2007), their large surface seems to hamper the targeting of the CK2 sites involved in the inhibitory activity, as observed for Mo_36_POM, indicating the importance of the shape complementarity between CK2 and the other tested POMs.

The structural analysis of the SAXS signal reveals the shape of the complex formed by CK2α and Ru_4_POM, with Ru_4_POM connecting two CK2α molecules, which, in turn, can interact with each other. Ru_4_POM binds to CK2α in a region adjacent to the ATP binding pocket, in between the hinge/αD region and the Gly-rich loop, overlapping part of the substrate-binding site. The two Ru_4_POM molecules do not directly interact to each other and bind CK2α through polar interactions, likely both hydrogen-bonds and electrostatic forces.

**FIGURE 10:**
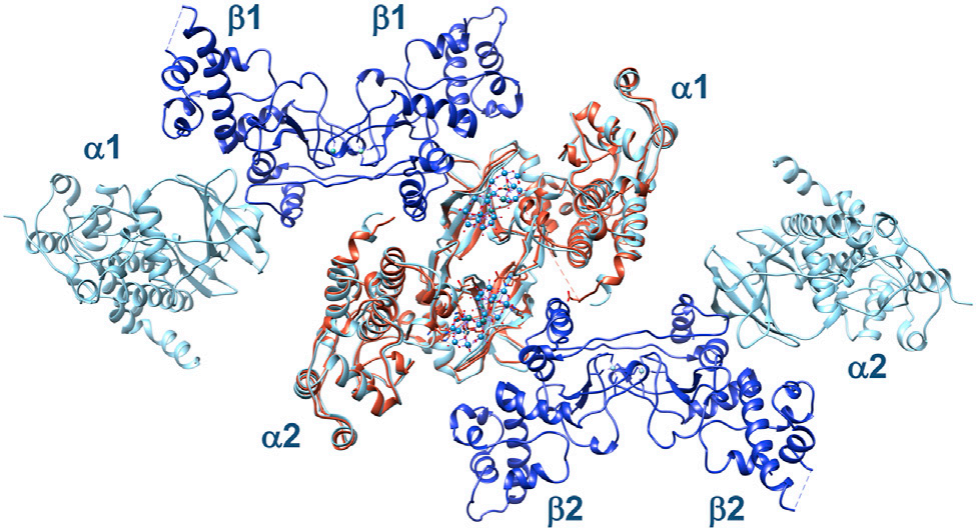
Structural superposition of two α_2_β_2_ tetramers, 1 and 2 (α-subunits in cyan, β-subunits in blue), on the (CK2α)_2_(Ru_4_POM)_2_ complex (in orange). In this arrangement, the two tetramers do not show evident steric clashes, suggesting that they can interact with Ru_4_POM in a similar way to the isolated catalytic subunit.

As Ru_4_POM is able to inhibit also the α_2_β_2_ holoenzyme, we structurally superposed two molecules of α_2_β_2_ on the (CK2α)_2_(Ru_4_POM)_2_ complex, to see whether the structural information derived by SAXS are valid also for the tetrameric holoenzyme (Figure 10). Indeed, we obtained a reasonable model for the hypothetical Ru_4_POM-mediated dimerization of the full enzyme, with no evident steric overlaps between the two tetramers. Then, we suggest that Ru_4_POM binds to α_2_β_2_ in a very similar way to that seen for the isolated CK2α, inhibiting the full enzyme with the same mechanism shown for the free catalytic subunit. It has been proposed that the mechanism of regulation of the enzyme relies on the self-inhibitory oligomerization of α_2_β_2_, mediated by inter-tetrameric electrostatic interactions involving the acidic loop of the β-subunit of one tetramer (Asp55–Asp64) and the positively charged P + 1 loop of the *α*-subunit of the other tetramer (Arg191–Lys198). It is plausible that Ru_4_POM is able to interfere with the normal regulation of the enzyme activity by affecting the oligomerization process of the tetrameric form of CK2. It is also possible that Ru_4_POM affect the interaction of CK2 with at least some of the interacting partners of this kinase.

In summary, we could unravel the mechanism of inhibition of a Ru-based POM, a compound stable in aqueous solution and able to inhibit CK2 in the very low nanomolar range. Cell internalization has also been addressed by using suitable transfecting agents. Ru_4_POM binds to CK2 and induces the formation of a (CK2α)_2_(Ru_4_POM)_2_ complex, which is inactive because the substrate-binding site and the ATP-binding site are inaccessible to substrates and ATP, respectively. This inhibition mechanism is entirely different from the common ATP-competitive one typical for most of the known protein kinase inhibitors. Ru_4_POM has physical and chemical properties very different from that of conventional kinase inhibitors (small organic molecules), and this, coupled to its ability of inhibition in cell-based assays, make this compound particularly interesting for further developments. Notably, Ru_4_POM is the first non-peptide molecule showing a substrate-competitive mechanism of inhibition. Atomic details of the interaction between CK2 and Ru_4_POM can be unravelled by the crystallographic analysis of the (CK2α)_2_(Ru_4_POM)_2_ complex, which would be useful to obtain information regarding the most relevant interactions that stabilize the complex. While many crystal structures of CK2 are available, alone or in complex with inhibitors (mainly ATP-competitive), complexes with substrate peptides have never been crystallized, suggesting that also the crystallization of CK2 in complex with POMs acting as substrate-competitive inhibitors could not be straightforward.

While a selectivity profile of Ru_4_POM against a protein kinase panel is beyond the purpose of this study, our findings suggest that a remarkable specificity for CK2 is highly conceivable, being this compound able to function as a substrate-competitive inhibitor of a kinase, CK2, with the uncommon property of targeting negatively charged substrates.

## Materials and Methods

### Recombinant CK2α Production

Recombinant full-length CK2α and tetrameric CK2 (α_2_β_2_) [produced as in (Venerando et al., 2013)] were kindly provided by Dr. Andrea Venerando (University of Parma, Italy). Human CK2α (residues 1-336) was produced as previously reported (Papinutto et al., 2012). Briefly, after expression in *E. coli* BL21-DE3, the protein was purified by two chromatographic steps, with heparin affinity chromatography (5 ml HiTrap Eparin HP column, GE Healthcare) and size-exclusion chromatography (HiLoad 16/60 Superdex 75 pg column, GE Healthcare). Protein eluted in 25 mM Tris, 500 mM NaCl, 1 mM DTT, pH = 8.5 was concentrated to 13.3 mg/ml, flash-frozen in liquid nitrogen and stored at −80°C.

### Polyoxometalates Synthesis and Characterization

Na_10_[Ru_4_(H_2_O)_4_(μ-O)_4_(μ-OH)_2_(γ-SiW_10_O_36_)_2_], Ru_4_POM, was prepared following a published procedure (Galiano et al., 2021). Ru_4_POM identity was confirmed by FTIR, while its stability was monitored by UV-vis, by using a Cary 100 instrument (Varian), in buffered aqueous environment (25 mM Tris, pH 8.5) over 24 h (Supplementary Figure S6).

(^*n*^Bu_4_N)_3_H[γ-SiW_10_O_36_{(C_5_H_7_N_2_OS)(CH_2_)_4_CONH(CH_2_)_3_Si}_2_O], Biotin-SiW_10_ (Zamolo et al., 2018), (*S*,*S*)-(^*n*^Bu_4_N)_4_[γ-SiW_10_O_36_{(C_16_H_9_)SO_2_NH(CH_2_)_3_Si}_2_O], Trp-SiW_10_ (Syrgiannis et al., 2019) and its (*R,R*) enantiomer, were prepared following previously reported procedures. The corresponding sodium salts were prepared by counterion exchange as described in (Zamolo et al., 2018). All hybrid POMs were characterized by FTIR and ESI-MS(-) to confirm their identity. (NH_4_)_12_[Mo_36_(NO)_4_O_108_(H_2_O)_16_], Mo_36_POM, was prepared as reported in (Amini et al., 2015).

### Analytical Size-Exclusion Chromatography

Analytical SEC analyses on isolated CK2α and on CK2α/Ru_4_POM mixtures were performed using a 10/30 Superdex 75 GL column (GE Healthcare) or a 10/30 Superdex 200 GL column (GE Healthcare) equilibrated with 25 mM Tris, 1 mM DTT, pH = 8.5 and NaCl at various concentrations, namely 0.5, 0.4, 0.3, and 0.2 M. For the preparation of CK2α/POM mixtures in different ratios, a concentrated stock solution of Ru_4_POM (5 mM) obtained by solubilizing dry powder in 25 mM Tris, 0.5 M NaCl, 1 mM DTT, pH = 8.5 was used. CK2α concentration varied from 200 to 300 μM, in accordance with SEC-SAXS experiments (see below).

### Isothermal Titration Calorimetry

Isothermal titration calorimetry (ITC) experiments were performed using a Malvern PEAQ-ITC microcalorimeter, at 25°C, with a 270 μl sample cell and a computer controlled microsyringe for titrant injections. CK2α (20–30 μM) and Ru_4_POM (200–400 μM) samples were in 25 mM Tris, 500 mM NaCl, pH = 8.5. After baseline stabilization, a further delay of 60 s was used before the first injection. In each individual titration, a starting Ru_4_POM injection of 0.4 μl in 0.8 s was followed by other 12 injections of 3 μl with a duration of 6 s each. A delay of 150 s was applied between each Ru_4_POM injection. Wiseman plot of integrated data was automatically obtained by software analysis (excluding the heat referred to the first 0.4 μl injection) and then it was fitted by theoretical binding curve using the one site model.

### SAXS Data Collection and Analysis

SAXS experiments were performed at the bio-SAXS beamline BM29 at ESRF, Grenoble, France (Pernot et al., 2013). The CK2α and CK2α/Ru_4_POM mixtures were measured by SEC-SAXS approaches (Brennich et al., 2017). Samples containing 300 μM Ru_4_POM, 300 μM CK2α and 300 μM CK2α:Ru_4_POM at 1:1 M ratio in buffer 25 mM Tris, 1 mM DTT, pH 8.5 and 0.5 M NaCl were loaded on a GE Superdex Increase 200 (3.2/300) column. For samples measured at 0.3 and 0.2 M NaCl (buffer 25 mM Tris, 1 mM DTT, pH 8.5) CK2α concentration was 130 μM. Samples with 200 μM CK2α with CK2α:Ru_4_POM at 1:2 and at 1:10 M ratio in buffer 25 mM Tris, 1 mM DTT, pH 8.5 and 0.5 M NaCl were loaded on a Agilent AdvanceBio SEC (4.6/300) and on a GE Superose 6 Increase (3.2/300) columns, respectively. The samples were centrifuged (16,000 g for 30 min at 4°C) prior the measurements and 50 μl of sample were injected in the columns. The purifications were carried-out *via* a high-performance liquid chromatography device (HPLC, Shimadzu) attached directly to the sample-inlet valve of the BM29 sample changer. The columns were equilibrated with 3 CV to obtain a stable background signal before measurement. A flow rate of 0.3 ml/min was used for all sample measurements. All SAXS data were collected at a wavelength of 0.99 Å using a sample-to-detector (PILATUS 2 M, DECTRIS) distance of 2.867 m. Data reduction and preliminary data processing were performed automatically using the Dahu/FreeSAS pipeline implemented at BM29. In the SEC-SAXS chromatograms, frames in regions of stable *R*_*g*_ were selected with CHROMIXS and averaged using PRIMUS to yield a single averaged frame for each protein sample. Analysis of the overall parameters was carried out by PRIMUS from ATSAS 3.0.4 package (Manalastas-Cantos et al., 2021). For CK2α and CK2α:Ru_4_POM at 1:1 the pair distance distribution functions, *P*(r), were used to calculate *ab initio* models in P1 and P2 symmetries, respectively, with DAMMIF (Manalastas-Cantos et al., 2021). Plots and protein models were generated using OriginPro 9.0 and UCSF Chimera software, respectively. SAXS parameters for data collection and analysis are summarized in Supplementary Table S1 following international guidelines (Trewhella et al., 2017). SAXS data were deposited into the Small Angle Scattering Biological Data Bank (SASBDB) under accession numbers SASDNF7 for CK2α and SASDNG7 for CK2α:Ru_4_POM 1:1.

### SAXS-Based Modelling of CK2α-Polyoxometalate Complex

The molecular docking of Ru_4_POM to CK2α was obtained exploiting Patchdock (Schneidman-Duhovny et al., 2005) using the pdb model of Ru_4_POM as ligand and CK2α (PDB 3q04) as receptor. The docking resulted in 19 plausible models of a complex formed by CK2α monomer binding a single molecule of Ru_4_POM bound in different positions along the entire positively charged cavity of CK2α. Symmdock (Schneidman-Duhovny et al., 2005) was then used to generate CK2α-Ru_4_POM dimers using as input the 19 plausible docking models for a total of about 1,900 dimeric species (i.e., the first 100 best scored dimers obtained with Symmdock per each of the 19 docking models obtained with Patchdock). Our data showed that CK2α never forms dimers or multimers in solution while Ru_4_POM induces CK2α dimerization at 1:1 M ratio. Based on this experimental observation, only the Symmdock-generated CK2α:Ru_4_POM dimers, where Ru_4_POM mediates intermolecular contacts between two CK2α monomer, were selected. The structures of models were ranked according to the lower χ^2^ value obtained by structure comparison between the models and the SAXS data of the CK2α-POM complex using CRYSOL (Manalastas-Cantos et al., 2021). The best model was then fitted into the DAMMIF envelope using SUPCOMB (Kozin and Svergun, 2001).

### *In Vitro* CK2 Activity Assay

For IC_50_ (concentrations inducing 50% inhibition) analysis, recombinant tetrameric CK2 (α_2_β_2_), or C-terminal-deleted CK2α (CK2α^1–336^), or full length CK2α (7.5 ng) were incubated with 0.1 mM synthetic peptide substrate RRRADDSDDDDD (CK2-tide), in a phosphorylation buffer containing 50 mM Tris-HCl pH 7.5, 10 mM MgCl_2_, 20 μM [γ-^33^P]ATP (1,000–2,000 cpm/pmol), in a final volume of 20 μl, with increasing concentrations of the inhibitor. In the case of tetrameric CK2, 0.1 M NaCl was also present in the phosphorylation mixture. Controls were performed in the absence of any inhibitor, but with equal volume of vehicle (H_2_O). Reactions were performed at 30°C for 12 min and stopped by sample absorption on phospho-cellulose papers. Papers were washed three times with 75 mM phosphoric acid and counted in a scintillation counter (PerkinElmer). In the case of casein substrate (used at 0.05 mg/ml concentration), reactions were stopped by the addition of Laemmli loading buffer, samples were analyzed by 11% SDS-PAGE, and radioactive bands were detected and quantified by digital autoradiography (Cyclone plus storage phosphor system, PerkinElmer). IC_50_ calculation was obtained by analysis of the results with GraphPad Prism 7.0a software.

For kinetics analysis, the assays were performed with increasing concentrations of CK2-tide or casein, in the phosphorylation buffer described above, but with 100 μM ATP concentration.

### Cell Culture, Treatments, and Lysis

HEK-293T cells (human embryo kidney fibroblasts) were cultured in an atmosphere containing 5% CO_2_, maintained in DMEM medium (Sigma), supplemented with 10% (v/v) fetal bovine serum (FBS), 2 mM L-glutamine, 100 U/ml penicillin, and 100 mg/ml streptomycin. Cell treatments with Ru_4_POM were performed in the culture medium. When used, the cationic transfection reagents PEI (Thermo Scientific) or JetOpitmus (Polyplus-transfection) were mixed with Ru_4_POM and incubated for 1 h at room temperature, before adding the mix to the cells. After 24 h cells were lysed as previously described (Zanin et al., 2012). Protein concentration was determined by the Bradford method.

### Endo-Cellular CK2 Activity Assay

Endo-cellular CK2 activity was evaluated by assessing the phosphorylation state of the CK2 substrate Akt phospho-Ser129 (Abcam) (Ruzzene et al., 2010). For this purpose, equal amounts of proteins from treated cells were loaded on 11% SDS-PAGE, blotted on Immobilon-P membranes (Millipore), processed in Western blot (WB), and detected by chemiluminescence.

Quantitation of the signal was obtained by chemiluminescence detection on ImageQuant LAS 500 (GE Healthcare Life Sciences) and analysis with Carestream Molecular Imaging software (Carestream).

### Statistical Analysis for Kinase Activity

Statistical significance was evaluated by One-way Anova analysis using GraphPad Prism 7 program. All values are expressed as means ± SEM. Comparisons of more than two groups were made with a one-way ANOVA using post-hoc Bonferroni’s test. Comparison of two groups was obtained by the Student’s t-test for unpaired data when appropriate. Differences were considered statistically significant at values of *p* < 0.05.

## Data Availability

The datasets presented in this study can be found in online repositories. The names of the repository/repositories and accession number(s) can be found in the article/Supplementary Material.

## References

[B1] Amini M. Naslhajian H. Farnia S. M. F. Hołyńska M. (2015). Selective Oxidation of Sulfides Catalyzed by the Nanocluster Polyoxomolybdate (NH_4_)_12_ [Mo_36_ (NO)_4_ O_108_ (H_2_ O)_16_ ]. Eur. J. Inorg. Chem. 2015, 3873–3878. 10.1002/ejic.201500528

[B2] Arefian M. Mirzaei M. Eshtiagh-Hosseini H. Frontera A. (2017). A Survey of the Different Roles of Polyoxometalates in Their Interaction with Amino Acids, Peptides and Proteins. Dalton Trans. 46, 6812–6829. 10.1039/c7dt00894e 28530742

[B3] Atkinson E. L. Iegre J. Brear P. D. Zhabina E. A. Hyvönen M. Spring D. R. (2021). Downfalls of Chemical Probes Acting at the Kinase ATP-Site: CK2 as a Case Study. Molecules 26, 1977. 10.3390/molecules26071977 33807474 PMC8037657

[B4] Battistutta R. Cozza G. Pierre F. Papinutto E. Lolli G. Sarno S. (2011). Unprecedented Selectivity and Structural Determinants of a New Class of Protein Kinase CK2 Inhibitors in Clinical Trials for the Treatment of Cancer. Biochemistry 50, 8478–8488. 10.1021/bi2008382 21870818

[B5] Battistutta R. Lolli G. (2011). Structural and Functional Determinants of Protein Kinase CK2α: Facts and Open Questions. Mol. Cell. Biochem. 356, 67–73. 10.1007/s11010-011-0939-6 21739155

[B6] Battistutta R. Mazzorana M. Cendron L. Bortolato A. Sarno S. Kazimierczuk Z. (2007). The ATP-Binding Site of Protein Kinase CK2 Holds a Positive Electrostatic Area and Conserved Water Molecules. Chembiochem 8, 1804–1809. 10.1002/cbic.200700307 17768728

[B7] Battistutta R. (2009). Protein Kinase CK2 in Health and Disease. Cell. Mol. Life Sci. 66, 1868–1889. 10.1007/s00018-009-9155-x 19387547 PMC11115547

[B8] Battistutta R. Sarno S. De Moliner E. Papinutto E. Zanotti G. Pinna L. A. (2000). The Replacement of ATP by the Competitive Inhibitor Emodin Induces Conformational Modifications in the Catalytic Site of Protein Kinase CK2. J. Biol. Chem. 275, 29618–29622. 10.1074/jbc.M004257200 10882732

[B9] Bijelic A. Aureliano M. Rompel A. (2019). Polyoxometalates as Potential Next‐Generation Metallodrugs in the Combat against Cancer. Angew. Chem. Int. Ed. 58, 2980–2999. 10.1002/anie.201803868 PMC6391951 29893459

[B10] Bonchio M. Syrgiannis Z. Burian M. Marino N. Pizzolato E. Dirian K. (2019). Hierarchical Organization of Perylene Bisimides and Polyoxometalates for Photo-Assisted Water Oxidation. Nat. Chem. 11, 146–153. 10.1038/s41557-018-0172-y 30510216

[B11] Borgo C. D’Amore C. Cesaro L. Sarno S. Pinna L. A. Ruzzene M. (2021a). How Can a Traffic Light Properly Work if it Is Always Green? the Paradox of CK2 Signaling. Crit. Rev. Biochem. Mol. Biol. 56, 1–39. 10.1080/10409238.2021.1908951 33843388

[B12] Borgo C. D’Amore C. Sarno S. Salvi M. Ruzzene M. (2021b). Protein Kinase CK2: a Potential Therapeutic Target for Diverse Human Diseases. Sig Transduct. Target Ther. 6, 183. 10.1038/s41392-021-00567-7 PMC8126563 33994545

[B13] Borgo C. Ruzzene M. (2021). “Protein Kinase CK2 Inhibition as a Pharmacological Strategy,” in Advances in Protein Chemistry and Structural Biology (San Diego, USA: Academic Press), 23–46. 10.1016/bs.apcsb.2020.09.003 33632467

[B14] Borgo C. Ruzzene M. (2019). Role of Protein Kinase CK2 in Antitumor Drug Resistance. J. Exp. Clin. Cancer Res. 38, 287. 10.1186/s13046-019-1292-y 31277672 PMC6612148

[B15] Brear P. Ball D. Stott K. D’Arcy S. Hyvönen M. (2020). Proposed Allosteric Inhibitors Bind to the ATP Site of CK2α. J. Med. Chem. 63, 12786–12798. 10.1021/acs.jmedchem.0c01173 33119282 PMC7666092

[B16] Brear P. De Fusco C. Hadje Georgiou K. Francis-Newton N. J. Stubbs C. J. Sore H. F. (2016). Specific Inhibition of CK2α from an Anchor outside the Active Site. Chem. Sci. 7, 6839–6845. 10.1039/c6sc02335e 28451126 PMC5355960

[B17] Brennich M. E. Round A. R. Hutin S. (2017). Online Size-Exclusion and Ion-Exchange Chromatography on a SAXS Beamline. J. Vis. Exp. 10.3791/54861 PMC5409194 28117806

[B18] Chua M. M. J. Lee M. Dominguez I. (2017). Cancer-type Dependent Expression of CK2 Transcripts. PLoS ONE 12, e0188854. 10.1371/journal.pone.0188854 29206231 PMC5714396

[B19] Čolović M. B. Lacković M. Lalatović J. Mougharbel A. S. Kortz U. Krstić D. Z. (2020). Polyoxometalates in Biomedicine: Update and Overview. Curr. Med. Chem. 27, 362–379. 10.2174/0929867326666190827153532 31453779

[B20] Croce M. Conti S. Maake C. Patzke G. R. (2019). Nanocomposites of Polyoxometalates and Chitosan‐Based Polymers as Tuneable Anticancer Agents. Eur. J. Inorg. Chem. 2019, 348–356. 10.1002/ejic.201800268

[B21] Dalle Vedove A. Zonta F. Zanforlin E. Demitri N. Ribaudo G. Cazzanelli G. (2020). A Novel Class of Selective CK2 Inhibitors Targeting its Open Hinge Conformation. Eur. J. Med. Chem. 195, 112267. 10.1016/j.ejmech.2020.112267 32283296

[B22] De Moliner E. Moro S. Sarno S. Zagotto G. Zanotti G. Pinna L. A. (2003). Inhibition of Protein Kinase CK2 by Anthraquinone-Related Compounds. J. Biol. Chem. 278, 1831–1836. 10.1074/jbc.M209367200 12419810

[B23] Dominguez I. Cruz-Gamero J. M. Corasolla V. Dacher N. Rangasamy S. Urbani A. (2021). Okur-Chung Neurodevelopmental Syndrome-Linked CK2α Variants Have Reduced Kinase Activity. Hum. Genet. 140, 1077–1096. 10.1007/s00439-021-02280-5 33944995

[B24] Duncan J. S. Litchfield D. W. (2008). Too Much of a Good Thing: the Role of Protein Kinase CK2 in Tumorigenesis and Prospects for Therapeutic Inhibition of CK2. Biochimica Biophysica Acta (BBA) - Proteins Proteomics 1784, 33–47. 10.1016/j.bbapap.2007.08.017 17931986

[B25] Ermakova I. Boldyreff B. Issinger O.-G. Niefind K. (2003). Crystal Structure of a C-Terminal Deletion Mutant of Human Protein Kinase CK2 Catalytic Subunit. J. Mol. Biol. 330, 925–934. 10.1016/s0022-2836(03)00638-7 12860116

[B26] Galiano F. Mancuso R. Carraro M. Bundschuh J. Hoinkis J. Bonchio M. (2021). A Polyoxometalate-Based Self-Cleaning Smart Material with Oxygenic Activity for Water Remediation with Membrane Technology. Appl. Mater. Today 23, 101002. 10.1016/j.apmt.2021.101002

[B27] Gobbo P. Tian L. Pavan Kumar B. V. V. S. Turvey S. Cattelan M. Patil A. J. (2020). Catalytic Processing in Ruthenium-Based Polyoxometalate Coacervate Protocells. Nat. Commun. 11, 41. 10.1038/s41467-019-13759-1 31900396 PMC6941959

[B28] Iegre J. Atkinson E. L. Brear P. D. Cooper B. M. Hyvönen M. Spring D. R. (2021). Chemical Probes Targeting the Kinase CK2: a Journey outside the Catalytic Box. Org. Biomol. Chem. 19, 4380–4396. 10.1039/d1ob00257k 34037044

[B29] Iegre J. Brear P. De Fusco C. Yoshida M. Mitchell S. L. Rossmann M. (2018). Second-generation CK2α Inhibitors Targeting the αD Pocket. Chem. Sci. 9, 3041–3049. 10.1039/c7sc05122k 29732088 PMC5916021

[B30] Jurrus E. Engel D. Star K. Monson K. Brandi J. Felberg L. E. (2018). Improvements to the APBS Biomolecular Solvation Software Suite. Protein Sci. 27, 112–128. 10.1002/pro.3280 28836357 PMC5734301

[B31] Kozin M. B. Svergun D. I. (2001). Automated Matching of High- and Low-Resolution Structural Models. J. Appl. Cryst. 34, 33–41. 10.1107/S0021889800014126

[B32] Lolli G. Battistutta R. (2015). “Structural Basis of CK2 Regulation by Autoinhibitory Oligomerization,” in *Protein Kinase CK2 Cellular Function In Normal and Disease States* Advances in Biochemistry in Health and Disease. Editors Ahmed K. Issinger O.-G. Szyszka R. (Dordrecht, NL: Springer International Publishing), 35–47. 10.1007/978-3-319-14544-0_3

[B33] Lolli G. Naressi D. Sarno S. Battistutta R. (2017). Characterization of the Oligomeric States of the CK2 α2β2 Holoenzyme in Solution. Biochem. J. 474, 2405–2416. 10.1042/BCJ20170189 28572157

[B34] Lolli G. Pinna L. A. Battistutta R. (2012). Structural Determinants of Protein Kinase CK2 Regulation by Autoinhibitory Polymerization. ACS Chem. Biol. 7, 1158–1163. 10.1021/cb300054n 22506723

[B35] Lolli G. Ranchio A. Battistutta R. (2014). Active Form of the Protein Kinase CK2 α2β2 Holoenzyme Is a Strong Complex with Symmetric Architecture. ACS Chem. Biol. 9, 366–371. 10.1021/cb400771y 24175891

[B36] Long D.-L. Tsunashima R. Cronin L. (2010). Polyoxometalates: Building Blocks for Functional Nanoscale Systems. Angew. Chem. Int. Ed. 49, 1736–1758. 10.1002/anie.200902483 20131346

[B37] Manalastas-Cantos K. Konarev P. V. Hajizadeh N. R. Kikhney A. G. Petoukhov M. V. Molodenskiy D. S. (2021). ATSAS 3.0: Expanded Functionality and New Tools for Small-Angle Scattering Data Analysis. J. Appl. Cryst. 54, 343–355. 10.1107/S1600576720013412 33833657 PMC7941305

[B38] Mazzorana M. Pinna L. A. Battistutta R. (2008). A Structural Insight into CK2 Inhibition. Mol. Cell. Biochem. 316, 57–62. 10.1007/s11010-008-9822-5 18626746

[B39] Meggio F. Pinna L. A. Marchiori F. Borin G. (1983). Polyglutamyl Peptides: a New Class of Inhibitors of Type-2 Casein Kinases. FEBS Lett. 162, 235–238. 10.1016/0014-5793(83)80762-5 6195015

[B40] Montazeri K. Bellmunt J. (2020). Erdafitinib for the Treatment of Metastatic Bladder Cancer. Expert Rev. Clin. Pharmacol. 13, 1–6. 10.1080/17512433.2020.1702025 31810398

[B41] Niefind K. Battistutta R. (2013). “Structural Bases of Protein Kinase CK2 Function and Inhibition,” in Protein Kinase CK2 (Oxford, UK: John Wiley & Sons), 1–75. 10.1002/9781118482490.ch1

[B42] Niefind K. Guerra B. Ermakowa I. Issinger O.-G. (2001). Crystal Structure of Human Protein Kinase CK2: Insights into Basic Properties of the CK2 Holoenzyme. EMBO J. 20, 5320–5331. 10.1093/emboj/20.19.5320 11574463 PMC125641

[B43] Niefind K. Guerra B. Ermakowa I. Issinger O.-G. (2000). Crystallization and Preliminary Characterization of Crystals of Human Protein Kinase CK2. Acta Crystallogr. D. Biol. Cryst. 56, 1680–1684. 10.1107/S0907444900013627 11092945

[B44] Niefind K. Guerra B. Pinna L. A. Issinger O. G. Schomburg D. (1998). Crystal Structure of the Catalytic Subunit of Protein Kinase CK2 from Zea mays at 2.1. Aresolution. EMBO J. 17, 2451–2462. 10.1093/emboj/17.9.2451 9564028 PMC1170587

[B45] Ortega C. E. Seidner Y. Dominguez I. (2014). Mining CK2 in Cancer. PLoS ONE 9, e115609. 10.1371/journal.pone.0115609 25541719 PMC4277308

[B46] Papinutto E. Ranchio A. Lolli G. Pinna L. A. Battistutta R. (2012). Structural and Functional Analysis of the Flexible Regions of the Catalytic α-subunit of Protein Kinase CK2. J. Struct. Biol. 177, 382–391. 10.1016/j.jsb.2011.12.007 22186626

[B47] Pernot P. Round A. Barrett R. De Maria Antolinos A. Gobbo A. Gordon E. (2013). Upgraded ESRF BM29 Beamline for SAXS on Macromolecules in Solution. J. Synchrotron Radiat. 20, 660–664. 10.1107/S0909049513010431 23765312 PMC3943554

[B48] Pinna L. A. (2002). Protein Kinase CK2: a Challenge to Canons. J. Cell. Sci. 115, 3873–3878. 10.1242/jcs.00074 12244125

[B49] Prudent R. Cochet C. (2009). New Protein Kinase CK2 Inhibitors: Jumping Out of the Catalytic Box. Chem. Biol. 16, 112–120. 10.1016/j.chembiol.2009.01.004 19246001

[B50] Prudent R. Moucadel V. Laudet B. Barette C. Lafanechère L. Hasenknopf B. (2008). Identification of Polyoxometalates as Nanomolar Noncompetitive Inhibitors of Protein Kinase CK2. Chem. Biol. 15, 683–692. 10.1016/j.chembiol.2008.05.018 18635005

[B51] Qiao Y. Chen T. Yang H. Chen Y. Lin H. Qu W. (2019). Small Molecule Modulators Targeting Protein Kinase CK1 and CK2. Eur. J. Med. Chem. 181, 111581. 10.1016/j.ejmech.2019.111581 31400711

[B52] Ramezani-Aliakbari M. Varshosaz J. Sadeghi-Aliabadi H. Hassanzadeh F. Rostami M. (2021). Biotin-Targeted Nanomicellar Formulation of an Anderson-Type Polyoxomolybdate: Synthesis and *In Vitro* Cytotoxicity Evaluations. Langmuir 37, 6475–6489. 10.1021/acs.langmuir.1c00623 34010005

[B53] Rodríguez F. Allende C. C. Allende J. E. (2005). Protein Kinase Casein Kinase 2 Holoenzyme Produced Ectopically in Human Cells Can Be Exported to the External Side of the Cellular Membrane. Proc. Natl. Acad. Sci. U.S.A. 102, 4718–4723. 10.1073/pnas.0501074102 15774585 PMC555726

[B54] Roskoski R. (2021). Properties of FDA-Approved Small Molecule Protein Kinase Inhibitors: A 2021 Update. Pharmacol. Res. 165, 105463. 10.1016/j.phrs.2021.105463 33513356

[B55] Ruzzene M. Di Maira G. Tosoni K. Pinna L. A. (2010). Assessment of CK2 Constitutive Activity in Cancer Cells. Meth. Enzymol. 484, 495–514. 10.1016/B978-0-12-381298-8.00024-1 21036247

[B56] Ruzzene M. Pinna L. A. (2010). Addiction to Protein Kinase CK2: a Common Denominator of Diverse Cancer Cells? Biochimica Biophysica Acta (BBA) - Proteins Proteomics 1804, 499–504. 10.1016/j.bbapap.2009.07.018 19665589

[B57] Sarno S. Papinutto E. Franchin C. Bain J. Elliott M. Meggio F. (2011). ATP Site-Directed Inhibitors of Protein Kinase CK2: an Update. Ctmc 11, 1340–1351. 10.2174/156802611795589638 21513497

[B58] Schneidman-Duhovny D. Inbar Y. Nussinov R. Wolfson H. J. (2005). PatchDock and SymmDock: Servers for Rigid and Symmetric Docking. Nucleic Acids Res. 33, W363–W367. 10.1093/nar/gki481 15980490 PMC1160241

[B59] Seetoh W.-G. Chan D. S.-H. Matak-Vinković D. Abell C. (2016). Mass Spectrometry Reveals Protein Kinase CK2 High-Order Oligomerization via the Circular and Linear Assembly. ACS Chem. Biol. 11, 1511–1517. 10.1021/acschembio.6b00064 26999075 PMC5868725

[B60] St-Denis N. A. Litchfield D. W. (2009). Protein Kinase CK2 in Health and Disease. Cell. Mol. Life Sci. 66, 1817–1829. 10.1007/s00018-009-9150-2 19387552 PMC11115660

[B61] Syrgiannis Z. Trautwein G. Calvaresi M. Modugno G. Zerbetto F. Carraro M. (2019). Controlling Size‐Dispersion of Single Walled Carbon Nanotubes by Interaction with Polyoxometalates Armed with a Tryptophan Tweezer. Eur. J. Inorg. Chem. 2019, 374–379. 10.1002/ejic.201800660

[B62] Tagliavini V. Honisch C. Serratì S. Azzariti A. Bonchio M. Ruzza P. (2021). Enhancing the Biological Activity of Polyoxometalate-Peptide Nano-Fibrils by Spacer Design. RSC Adv. 11, 4952–4957. 10.1039/D0RA10218K 35424453 PMC8694496

[B63] Trembley J. H. Chen Z. Unger G. Slaton J. Kren B. T. Van Waes C. (2010). Emergence of Protein Kinase CK2 as a Key Target in Cancer Therapy. Biofactors 36, 187–195. 10.1002/biof.96 20533398 PMC2916697

[B64] Trewhella J. Duff A. P. Durand D. Gabel F. Guss J. M. Hendrickson W. A. (2017). 2017 Publication Guidelines for Structural Modelling of Small-Angle Scattering Data from Biomolecules in Solution: an Update. Acta Cryst. Sect. D. Struct. Biol. 73, 710–728. 10.1107/S2059798317011597 28876235 PMC5586245

[B65] Van Rompuy L. S. Parac-Vogt T. N. (2019). Interactions between Polyoxometalates and Biological Systems: from Drug Design to Artificial Enzymes. Curr. Opin. Biotechnol. 58, 92–99. 10.1016/j.copbio.2018.11.013 30529815

[B66] Vandebroek L. Noguchi H. Kamata K. Tame J. R. H. Van Meervelt L. Parac-Vogt T. N. (2021). Shape and Size Complementarity-Induced Formation of Supramolecular Protein Assemblies with Metal-Oxo Clusters. Cryst. Growth . Des. 21, 1307–1313. 10.1021/acs.cgd.0c01571

[B67] Venerando A. Franchin C. Cant N. Cozza G. Pagano M. A. Tosoni K. (2013). Detection of Phospho-Sites Generated by Protein Kinase CK2 in CFTR: Mechanistic Aspects of Thr1471 Phosphorylation. PLOS ONE 8, e74232. 10.1371/journal.pone.0074232 24058532 PMC3776838

[B68] Venerando A. Ruzzene M. Pinna L. A. (2014). Casein Kinase: The Triple Meaning of a Misnomer. Biochem. J. 460, 141–156. 10.1042/BJ20140178 24825444

[B69] Wang S.-S. Yang G.-Y. (2015). Recent Advances in Polyoxometalate-Catalyzed Reactions. Chem. Rev. 115, 4893–4962. 10.1021/cr500390v 25965251

[B70] Zamolo V. A. Modugno G. Lubian E. Cazzolaro A. Mancin F. Giotta L. (2018). Selective Targeting of Proteins by Hybrid Polyoxometalates: Interaction between a Bis-Biotinylated Hybrid Conjugate and Avidin. Front. Chem. 6, 278. 10.3389/fchem.2018.00278 30050897 PMC6050359

[B71] Zanin S. Borgo C. Girardi C. O'Brien S. E. Miyata Y. Pinna L. A. (2012). Effects of the CK2 Inhibitors CX-4945 and CX-5011 on Drug-Resistant Cells. PLoS ONE 7, e49193. 10.1371/journal.pone.0049193 23145120 PMC3493520

[B72] Zhang G. Keita B. Craescu C. T. Miron S. de Oliveira P. Nadjo L. (2008). Molecular Interactions between Wells−Dawson Type Polyoxometalates and Human Serum Albumin. Biomacromolecules 9, 812–817. 10.1021/bm701120j 18266320

[B73] Zhang G. Keita B. Craescu C. T. Miron S. de Oliveira P. Nadjo L. (2007). Polyoxometalate Binding to Human Serum Albumin: a Thermodynamic and Spectroscopic Approach. J. Phys. Chem. B 111, 11253–11259. 10.1021/jp072947u 17784743

[B74] Zhao M. Chen X. Chi G. Shuai D. Wang L. Chen B. (2020). Research Progress on the Inhibition of Enzymes by Polyoxometalates. Inorg. Chem. Front. 7, 4320–4332. 10.1039/D0QI00860E

