# Supplementary material for: Mechanism of CK2 Inhibition by a Ruthenium-Based Polyoxometalate
Source: Front Mol Biosci. 2022 Jun 2;9:906390. doi: 10.3389/fmolb.2022.906390 (PMC9201508; doi:10.3389/fmolb.2022.906390)
Supplement: Supplementary file 1 [file DataSheet1.PDF]

Mechanism of CK2 inhibition by a ruthenium-based polyoxometalate

Simone Fabbian[1], Gabriele Giachin[1], Massimo Bellanda[1,2], Christian Borgo[3], Maria Ruzzene[3,4*], Giacomo Spuri[3], Ambra Campofelice[1], Laura Veneziano[1], Marcella Bonchio[1,5], Mauro Carraro[1*], Roberto Battistutta[1,2*]

[1] Department of Chemical Sciences, University of Padova, via F. Marzolo 1 35131 Padova, ITALY

[2] CNR Institute of Biomolecular Chemistry, UoS of Padova, via F. Marzolo 1 35131 Padova, ITALY

[3] Department of Biomedical Sciences, University of Padova, via U. Bassi 58/B 35121 Padova, ITALY

[4] CNR Institute of Neurosciences, via U. Bassi 58/B 35121 Padova, ITALY

[5] ITM-CNR, UoS of Padova, via F. Marzolo 1 35131 Padova, ITALY

[*] Correspondence to Roberto Battistutta (roberto.battistutta@unipd.it; (+39) 049.827.5262), Maria Ruzzene (maria.ruzzene@unipd.it; (+39) 049.827.6112), Mauro Carraro (mauro.carraro@unipd.it; (+39) 049.827.5256).

SUPPLEMENTARY MATERIAL

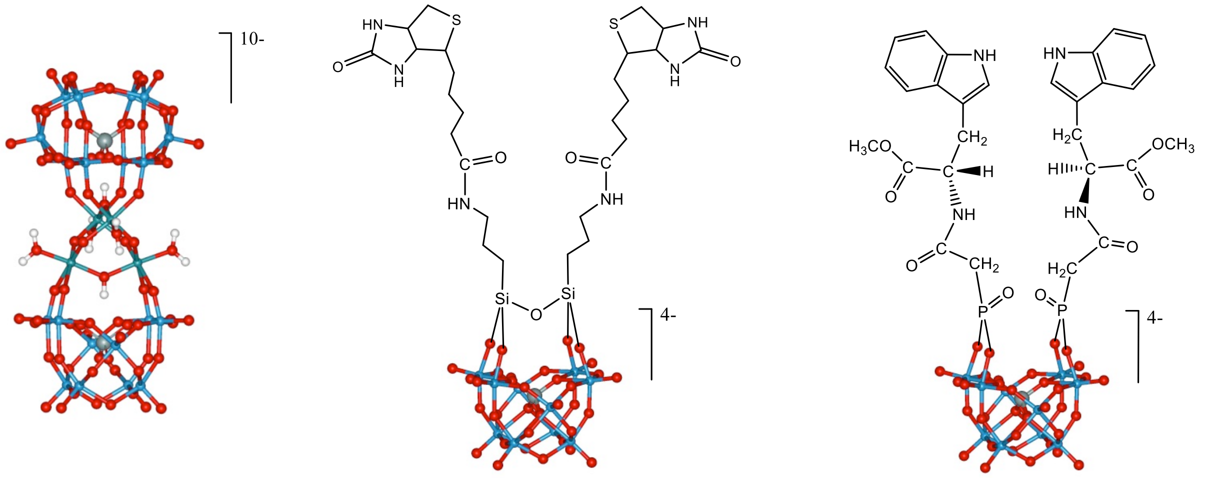

Suppl. Fig. 1. Structures of the POMs containing the subunit [$SiW_{10}O_{36}$] (with tungsten atoms in blue, oxygen atoms in red, silicon in grey, ruthenium atoms in green):

left, $[Ru_4(H_2O)_4(\mu\text{-}O)_4(\mu\text{-}OH)_2(\gamma\text{-}SiW_{10}O_{36})_2]^{10-}$ (Ru_4POM);

center, $[\gamma\text{-}SiW_{10}O_{36}\{(C_5H_7N_2OS)(CH_2)_4CONH(CH_2)_3Si\}_2O]^{4-}$ (Biotin-SiW_{10});

right, $[\gamma\text{-}SiW_{10}O_{36}\{(C_{16}H_9)SO_2NH(CH_2)_3Si\}_2O]^{4-}$ (Trp-SiW_{10}).

Tetrabutyl ammonium counterions have been omitted for clarity reasons, while only the (S,S) enantiomer of Trp-SiW_{10} is herein reported.

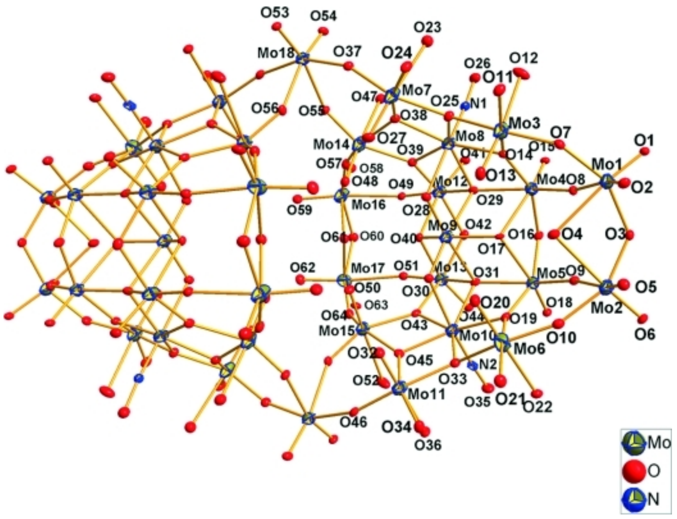

Suppl. Fig. 2. Crystallographic structure of $[Mo_{36}(NO)_4O_{108}(H_2O)_{16}]^{12-}$ (Mo_{36}POM) (with molybdenum atoms in blue and green; oxygen atoms in red, nitrogen atoms in blue) (Reproduced with permission of John Wiley & Sons Ltd from Amini M., et al., Eur. J. Inorg. Chem., 2015: 3873-3878).

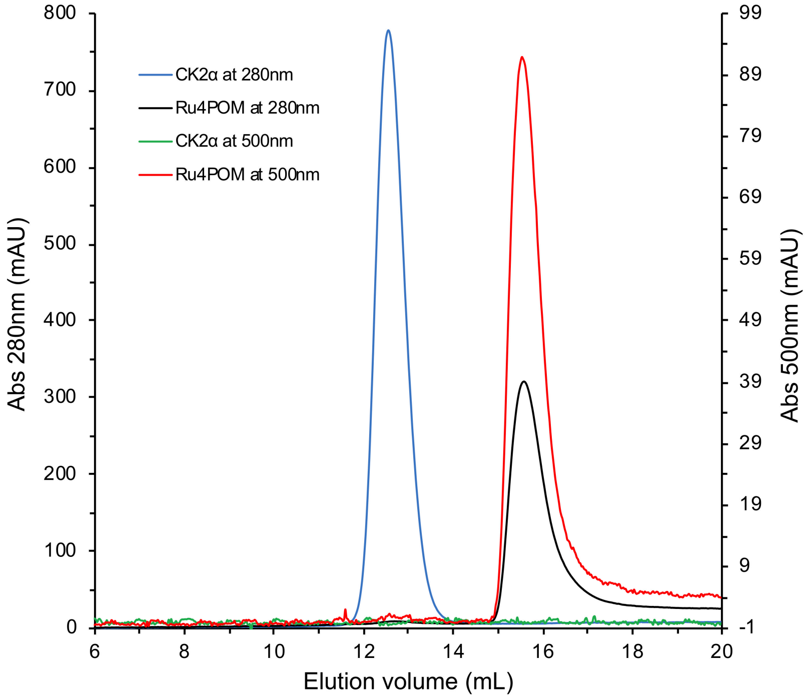

Suppl. Fig. 3. SEC elution profiles of CK2α and Ru_4POM. In 25 mM Tris, 500 mM NaCl, 1 mM DTT, pH = 8.5, CK2α elutes in monomeric form at 12.6 ml elution time (blue curve, Abs at 280 nm), on a 10/30 Superdex 75 GL column. As expected, no absorption was detected at 500 nm for CK2α (green curve). In the same elution system, Ru_4POM has an elution volume of 16.0 ml, in accordance with the lower dimensions. Ru_4POM is detected both at 500 nm, red curve, and at 280 nm, black curve.

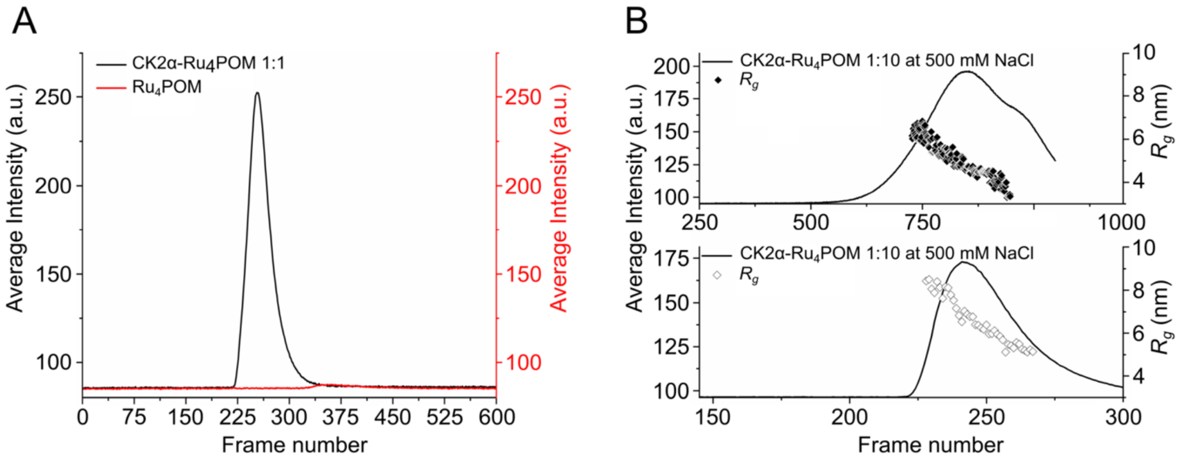

Suppl. Fig. 4. (A) Profiles for Ru4POM alone at 300 µM (red line) and for CK2α-Ru4POM 1:1 molar ratio (300 µM) showing negligible scattering contribution compared to the complex (black line). **(B)** SEC-SAXS chromatograms of CK2α-Ru4POM complexes formed at 1:10 and 1:2 molar ratio conditions (upper and lower panel, respectively).

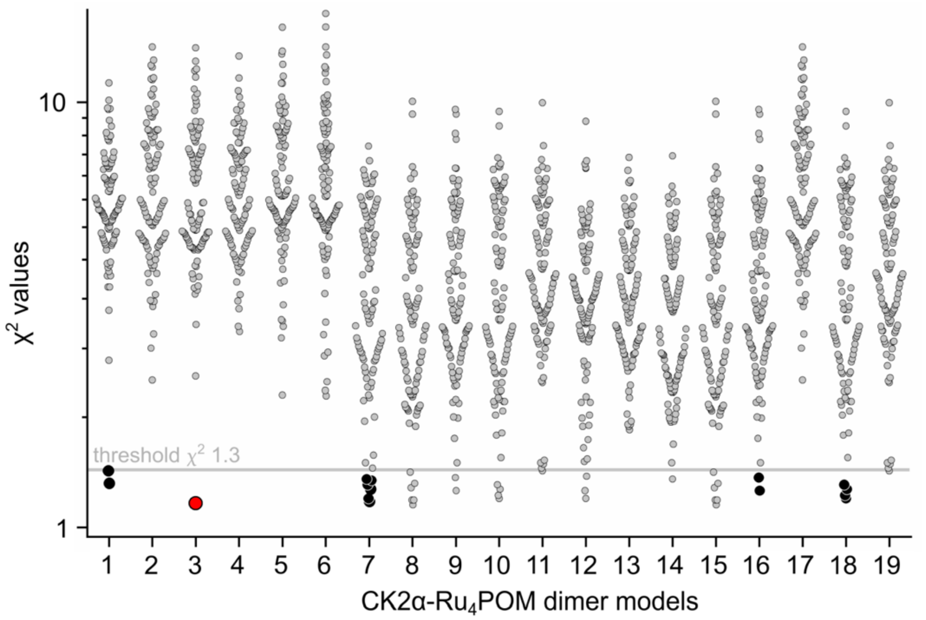

Suppl. Fig. 5. Scoring the best models according the best χ^2 values (here in log scale) obtained using CRYSOL. Each dot corresponds to a CK2α-Ru_4POM dimer for which the χ^2 value was calculated. Models with a χ^2 value below 1.3 (*i.e.* a reference χ^2 value obtained from SAXS CK2α data comparison to the corresponding X-ray crystal structure) were individually analyzed to identify dimers stabilized by inter-molecular Ru_4POM-mediated contacts (black dots). Red dot represents the best model in terms of CRYSOL χ^2 value (1.14) and fitting to DAMMIF envelope.

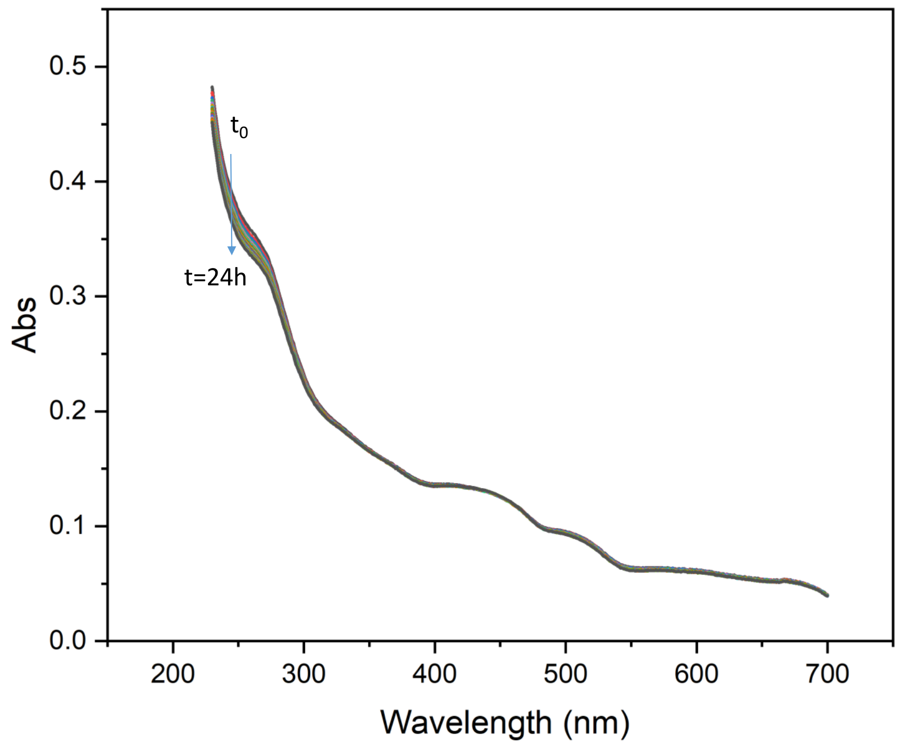

Suppl. Fig. 6. UV-vis spectrophotometric monitoring of Ru_4POM (5μM) stability in 25 mM Tris and 500 mM NaCl (pH=8.5) over time (up to 24 h). A quartz cuvette with path length 1 cm was used.

Suppl. Table 1. SAXS results for the proteins investigated in this study

a) Sample details

Sample name	CK2α	$(CK2\alpha)_2(Ru_4POM)_2$
Organism	*Homo sapiens sapiens*	
UniProt sequence ID (residues in construct)	CK2α (Casein kinase II subunit alpha), UniProt ID Q8NEV1 (3-330)	
Ligand	-	Ru_4POM: $Na_{10}[Ru_4(\mu\text{–}O)_4(\mu\text{–}OH)_2(H_2O)_4(\gamma\text{–}SiW_{10}O_{36})_2]$
Calculated molecular weight (Da)	39239	Monomer: 39239 (CK2α) + 5690 (Ru4POM) = 44929
Total frames (frames used for data analysis)	650 (265-300)	650 (255-275)
SEC column	GE Superdex Increase 200 (3.2/300)	
Injected volume (µL)	50	
Loading concentration (µM)	300	300
Flow rates (mL/min)	0.3	
SEC buffer	25 mM Tris, 500 mM NaCl, pH 8.5	

b) SAXS data collection parameters

Instrument	ESRF BM29
Wavelength (Å)	0.99
q-range (Å)	0.004-0.5
Sample-to-detector distance (m)	2.867
Exposure time	2 sec/frame
Temperature (° C)	20
Detector	Pilatus3 X 2M (Dectris)
Flux (photons/s)	2×10^{12}
Beam size (µm)	100 x 100
Sample configuration	1.8 mm quartz glass capillary
Absolute scaling method	Comparison to water in sample capillary
Normalization	To transmitted intensity by beam-stop counter

(c) Structural parameters

	CK2α	$(CK2\alpha)_2(Ru_4POM)_2$
**Guinier analysis**		
- I(0) (cm^{-1})	0.0844 ± 9.60E-05	0.0957 ± 1.68E-04
- R_g (nm)	2.23	3.09 ± 0.01
- *q*-range (nm^{-1}), point range	0.0151 – 0.3370, 6-96	0.0162 – 0.1740, 15-73
***P*(r) analysis**		
- I(0) (cm^{-1})	0.08416	0.096
- R_g (nm)	2.22	3.19

- D_{max} (nm)	6.7	11.8
- q-range (nm^{-1}), point range	0.0139 – 3.36, 2-678	0.0292 – 3.6, 12-680
- Porod volume (nm^3)	59.339	122.3
- χ^2 [total estimate from GNOM]	0.9232	0.8436
- Mass estimate based on volume (Da)	39559	81533
- Ratio mass estimated/expected	1	1.9

(d) Software employed for SAXS data reduction, analysis and interpretation

SAXS data reduction and data processing	EDNA and Primus (ATSAS 3.0.4)
Shape/bead modelling	DAMMIF (ATSAS 3.0.4)
3D graphic representation	UCSF Chimera 1.15
Docking and dimerization tools	Patchdock and Symmdock

(e) Shape model-fitting results and rigid-body atomistic modelling

	CK2α	$(CK2\alpha)_2(Ru_4POM)_2$
**DAMMIF**	*Default parameters, 20 calculation runs*	
- q-range (nm^{-1})	0.0051-4.5	0.0019-4.5
- Symmetry	P1	P2
- Normalized spatial discrepancy, σ	0.633, 0.019	1.9, 0.27
- χ^2 range values for the fitting	1.2	0.99
- Resolution (from SASRES, in Å)	20	50
**CRYSOL**	*Default parameters*	
- ID for rigid bodies	PDB: 3q04	SASBDB: SASDNG7
- Rigid bodies (residue numbers)	3-330	3-330
- χ^2 value for the fitting	1.29	1.14

(f) Small Angle Scattering Biological Data Bank (SASBDB)

SASBDB ID CK2α	SASDNF7<br>https://www.sasbdb.org/data/SASDNF7/gpfxay28sd/
SASBDB ID $(CK2\alpha)_2(Ru_4POM)_2$	SASDNG7<br>https://www.sasbdb.org/data/SASDNG7/c45jp02rdd/
